# Adverse outcome pathways of titanium dioxide nanoparticles and marine heatwaves on byssal performance in mussels

**DOI:** 10.1016/j.eehl.2026.100265

**Published:** 2026-07-17

**Authors:** Shuaishuai Wei, Zhihan Tu, Inna Sokolova, Liming Chen, Shixiu Wang, Menghong Hu, Youji Wang

**Affiliations:** a International Research Center for Marine Biosciences at Shanghai Ocean University, Ministry of Science and Technology, Shanghai Ocean University, Shanghai, 201306, China; b Key Laboratory of Exploration and Utilization of Aquatic Genetic Resources, Ministry of Education, Shanghai Ocean University, Shanghai, 201306, China; c Department of Marine Biology, Institute for Biological Sciences, University of Rostock, Rostock, Germany; d Department of Maritime Systems, Interdisciplinary Faculty, University of Rostock, Rostock, Germany; e Marine Biomedical Science and Technology Innovation Platform of Lin-gang Special Area, Shanghai, 201306, China

**Keywords:** *Mytilus coruscus*, Nano-TiO_2_, Byssal property, Cytotoxicity, Adverse outcome pathways

## Abstract

Adverse outcome pathways (AOPs) describe toxicity across levels of biological organization by measurable key events (KEs), thus providing a structured framework for predicting population-level effects. Here, mussels were exposed to different temperature conditions—22 °C and marine heatwave (MHW), in combination with nano-titanium dioxide (nano-TiO_2_) at 0, 25, and 250 μg/L. High-concentration nano-TiO_2_ and MHW exposure promoted the secretion of byssal threads and enhanced the adhesion to the substrate. However, they also caused severe histopathological damage in the foot and reduced the condition index (CI), indicating serious organism-level adverse outcomes (AOs). Accumulation of lipid peroxidation (LPO) products and redox imbalance suggested that MHW and nano-TiO_2_ induced oxidative stress, leading to increased cell apoptosis (CA), reduced cell viability (CV), and consequent tissue damage in the foot. Nano-TiO_2_ and MHW exposure significantly upregulated adhesion-related genes (*Mfp-3/5/6*) as well as immune- and cytotoxicity-related genes (*Caspase-3*, *TLR-4*, *Myd88*, and *TNF-α*). In contrast, *Mfp-1/2/4* and pre-collagen proteins (*preCOL-D/P/NG*) were significantly downregulated, resulting in poor extensibility of byssal threads. Using the AOP framework, this study links nano-TiO_2_ toxicity and MHW-induced stress to molecular disruptions, organism-level adverse outcomes, and altered mussel byssal defenses. These findings highlight that the interaction between MHW and nanoparticles may compromise mussel attachment and survival, with potential implications for the stability of coastal benthic ecosystems under future climate change scenarios.

## Highlights

• The effects of MHWs and nano-TiO_2_ to marine mussels on byssal performance were investigated.
• Oxidative stress initiates inflammation, apoptosis, and energy imbalance.
• AOP framework links molecular events to ecological outcomes under nano-TiO_2_ and MHWs exposure.

## Graphical abstract

**Figure:**
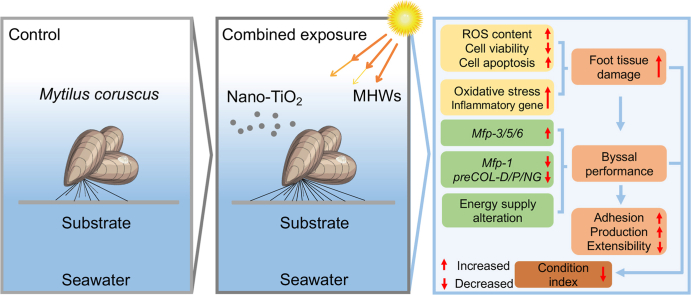

## 1 Introduction

Anthropogenic activities such as the use of fossil fuels have increased atmospheric carbon dioxide (CO_2_) concentrations, which results in global climate change [[1], [2], [3], [4]]. Climate change has led to an increase in the intensity and frequency of extreme weather events such as marine heatwaves (MHWs) over the past 40 years [5,6]. MHW can disrupt marine ecosystems by altering primary productivity, displacing habitats, changing the population dynamics of marine species, and increasing the frequency of fishery disasters [[7], [8], [9]]. While the physical drivers of MHW have been extensively studied [[10], [11], [12]], global assessments of their ecological impacts remain limited [13], particularly in relation to biological stress responses and the mechanisms driving mass mortality events. These knowledge gaps become even more critical when considering the compounding effects of emerging stressors such as nanopollution, which may interact with MHW to intensify physiological strain on vulnerable keystone species like marine mussels.

Through the formation of biogenic reefs, marine bivalves create complex three-dimensional habitats that support biodiversity, and provide critical ecosystem services such as shoreline protection and sediment stabilization [14,15]. The establishment and persistence of these biogenic reef systems rely heavily on effective attachment to the surrounding substrate. In mussels, attachment is mediated by byssal threads, specialized proteinaceous fibers that anchor individuals in high-energy coastal environments. These proteinaceous fibers play key roles in group cohesion within beds, enhancing resistance to wave action, promoting reef integrity, and offering protection from predation and dislodgement [16]. Moreover, the energetic and physiological investment in byssus production makes it particularly sensitive to environmental stressors [[17], [18], [19]], highlighting its relevance for assessing the impacts of climate extremes and pollution.

The byssus consists of a complex matrix of collagen-like proteins with specialized adhesive properties and comprises two main components: the byssal thread, which provides toughness and flexibility, and the adhesive plaque, which anchors the thread to substrates [20,21]. Increasing evidence indicates that pollutants and thermal stress can impair both the production and mechanical performance of the byssus [[22], [23], [24], [25], [26]]. Although the precise mechanisms remain unclear, one potential pathway involves disruption of redox balance, which is essential for byssus formation and functionality, particularly in protecting oxidation-sensitive components such as 3,4-dihydroxyphenylalanine (Dopa) [27,28]. Pollutants can alter the redox environment at adhesion sites, thereby weakening byssus performance [21,29]. For example, exposure to ferroferric oxide nanoparticles (nano-Fe_3_O_4_), zinc oxide nanoparticles (nano-ZnO), sodium hypochlorite (NaClO), and hydrogen peroxide (H_2_O_2_) has been shown to reduce both byssus production and adhesive capacity in mussels [25,29,30]. Moreover, environmental stressors such as pollutants and elevated temperatures can impair mussel redox physiology, compromising the synthesis of key proteins and the antioxidant defenses vital for proper byssus formation [27,28].

Nano-titanium dioxide (nano-TiO_2_) is an engineered nanomaterial used in cosmetics, medicine, and surface coatings [[31], [32], [33]]. Coastal marine ecosystems are an important sink for nano-TiO_2_ [34]. In shallow coastal zones, concentrations of nano-TiO_2_ can reach tens to hundreds of μg/L [[35], [36], [37]], owing to the release from sunscreens [37]. Metal oxide nanoparticles, including nano-TiO_2_, are toxic to marine organisms, including mussels, with oxidative stress recognized as a key mechanism of their toxicity [[38], [39], [40]]. Seasonally elevated temperatures can coincide with persistent nano-TiO_2_ pollution [37]. Thermal stress caused by MHW is known to elevate reactive oxygen species (ROS) production, overwhelm antioxidant defense systems, and lead to oxidative damage to DNA, proteins, and lipids [41]. This oxidative imbalance may be exacerbated by co-exposure to other pro-oxidants like nano-TiO_2_, potentially impairing mussel foot function and byssus formation. Although previous studies have indicated that ocean acidification [16,23] and sustained warming, particularly when combined with nano-TiO_2_ exposure [42], may impair mussel byssal defense performance, temperature in natural marine environments is highly variable (e.g., during MHW), and nanoparticle concentrations are also highly dynamic. The impacts of such realistic environmental fluctuations on mussel physiological performance, therefore, remain insufficiently understood, and the mechanistic pathways through which MHW and environmentally relevant concentrations of nano-TiO_2_ impact byssus-related processes remain largely unexplored.

To understand the interactions between multiple environmental stressors, tiered testing approaches are increasingly used to assess their biological effects [43]. The Adverse Outcome Pathways (AOPs) framework supports this by linking molecular and cellular key events (KEs) to adverse outcomes (AOs), aiding in the identification of toxicological mechanisms and informing risk assessments [[44], [45], [46], [47]]. In this study, we applied the AOP framework to investigate the combined effects of MHW and nano-TiO_2_ on byssus-related traits in *Mytilus coruscus*. At the KE level, we quantified disruptions in energy metabolism, including glucose, lipid (triglyceride), and albumin, and redox homeostasis in foot tissue. Concurrently, we explored molecular mechanisms by measuring the mRNA expression of the foot adhesion proteins (*Mfp-1/2/3/4/5/6*), pre-collagen proteins (*preCOL-D/P/NG*), and key components in the immune-related [toll-like receptor 4 (*TLR-4*), *Myd88*, and heat shock protein (*HSP*)*-70/90*] and cytotoxicity-related [tumor necrosis factor-α (*TNF-α*) and *Caspase-3*] signaling pathways. Cellular levels were also evaluated, focusing on cell viability (CV), oxidative stress markers such as ROS, and cell apoptosis (CA) in foot cells. The corresponding AOs evaluated in this study were tissue damage [assessed by the number of basophilic granulocytes (NBG), the length of the ciliated epithelium (LCE), and the proportion of foot gland area], impaired byssal thread production and adhesion, and a reduced condition index (CI). Our findings highlight the central role of redox imbalance in mediating the toxic effects of MHW and nano-TiO_2_, thereby improving predictions of climate and nanopollution impacts on this ecologically and economically important marine species.

## 2 Materials and methods

### 2.1 Preparation of animals and nano-TiO_2_

Adult *M. coruscus* (shell length: 78.53 ± 5.28 mm; wet mass: 35.61 ± 7.3 g) were sampled in Gouqi Island (30°43′14.268″N, 122°47′0.3696″E) in the East China Sea. Thirty liters of filtered artificial seawater were used in recirculating aquarium systems (240 L/h) to acclimate marine mussels at 22.1 ± 0.8 °C for two weeks. Environmental parameters, including pH (8.05 ± 0.02), salinity (24.9 ± 0.7), dissolved oxygen (5.7 ± 0.5 mg/L), and photoperiod (12 h light:12 h dark), were maintained to match the conditions at the sampling site, as described by Gu et al. [48]. Mussels were fed *Chlorella vulgaris* at approximately 5% of their dry weight, and seawater was renewed daily.

Anatase form of nano-TiO_2_ (25 nm, CAS: 13463-67-7) was obtained from Shanghai Maclin Biochemical Technology Co., Ltd. (Shanghai, China). Prior to use, a 2.5 g/L nano-TiO_2_ stock suspension was sonicated for 20 min (50 W, 40 kHz) using an ultrasonic processor (UP200S, Hielscher Ultrasonic Technology, Teltow, Germany) to minimize aggregation. The description of surface morphology, hydrodynamic diameter, zeta potential, and concentrations of nano-TiO_2_ in the exposure experiments is detailed in Text S1 and Table S1.

### 2.2 Temperature setting and experimental design

**Fig. 1:**
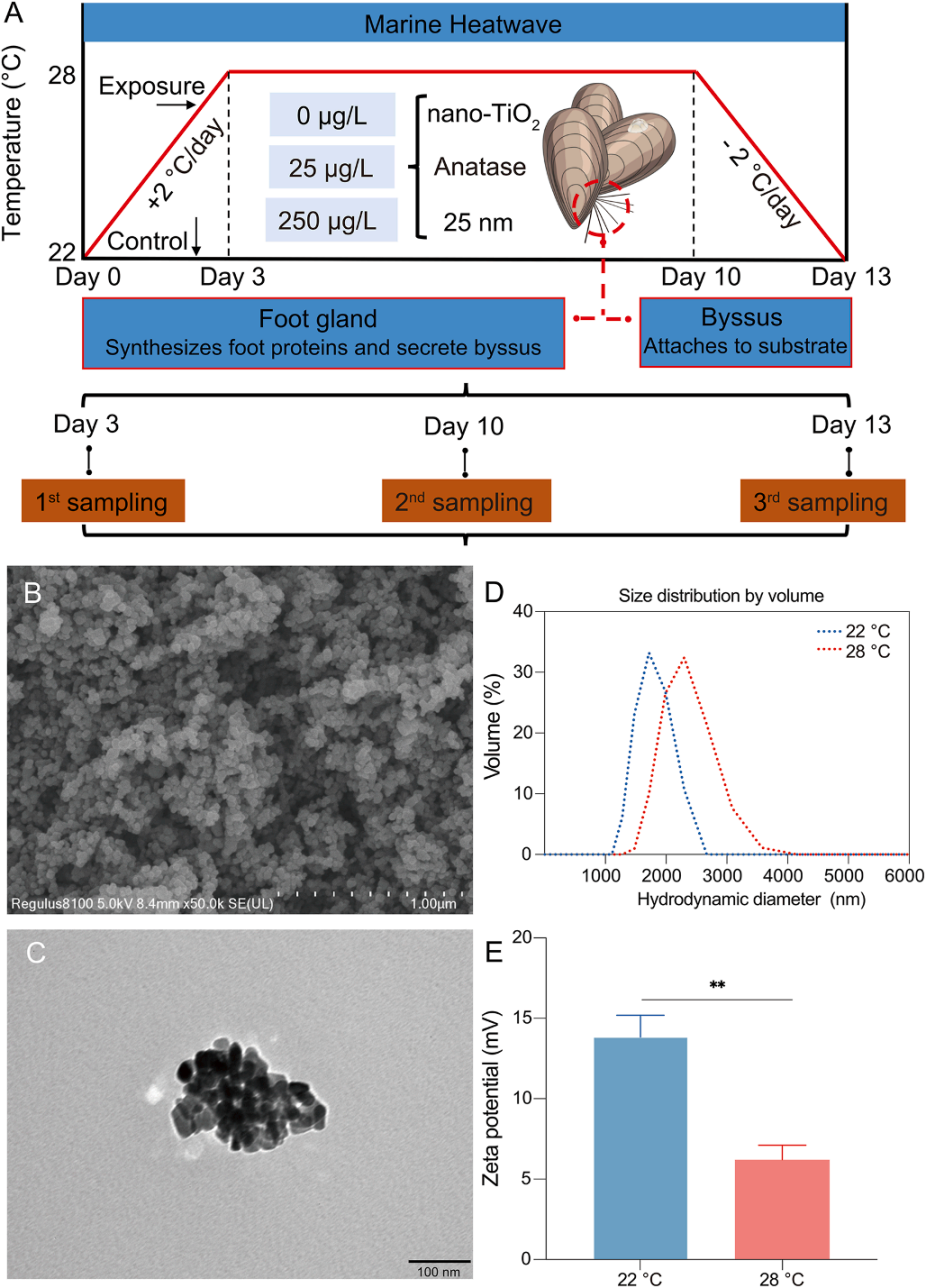
Experimental design and sampling points (A). Characterization of nano-TiO_2_, including SEM images (B), TEM images (C), dynamic light scattering (DLS) analysis at 22 °C and 28 °C (D), and zeta potential measurements at 22 °C and 28 °C (E). Asterisks (∗) denote significant differences between temperature regimes (∗*P* < 0.05, ∗∗*P* < 0.01).

As a marginal sea of the Pacific Ocean, the ECS has experienced increases in the frequency, intensity, and duration of MHW in recent years, with temperatures exceeding 28 °C for several consecutive days [[49], [50], [51]]. Accordingly, 22 °C was designated as the baseline (normal) temperature, based on regional climatological data [52], while 28 °C was selected to simulate extreme MHW conditions. Mussels were exposed for 13 days under two thermal regimes: (i) constant 22 °C for the normal temperature groups, and (ii) a gradual thermal increase from 22 °C to 28 °C over 3 days (2 °C/d), followed by 7 days at 28 °C and then a gradual return to 22 °C over 3 days for the MHW groups (Fig. 1A).

Environmental concentrations of nano-TiO_2_ in coastal waters have been reported to span 20–900 μg/L [35,37]. Based on this range, 25 μg/L was chosen as an ambient concentration [53], whereas 250 μg/L was designated as an environmentally relevant threshold. Six experimental groups were established using a fully crossed design with three concentrations of nano-TiO_2_ (control: 0 μg/L, ambient: 25 μg/L, and environmentally relevant threshold: 250 μg/L) crossed with two temperature regimes (control: 22 °C, and MHW: 22 °C→28 °C→22 °C). For brevity, the six treatments are hereafter abbreviated as CK (control; 0 μg/L, 22 °C), LT (low nano-TiO_2_; 25 μg/L, 22 °C), HT (high nano-TiO_2_; 250 μg/L, 22 °C), MHW (marine heatwave; 0 μg/L, MHW), LT-MHW (low nano-TiO_2_ + MHW; 25 μg/L, MHW), and HT-MHW (high nano-TiO_2_ + MHW; 250 μg/L, MHW).

A total of 30 glass aquaria (50 L each containing ten mussels) were used, with five replicate aquaria per treatment. Water temperatures were regulated using electronic thermostats (Loligo Systems ApS, Tjele, Denmark). Seawater was replaced daily with fresh seawater pre-adjusted to the corresponding exposure temperatures. Continuous aeration was applied to the exposure media to reduce nano-TiO_2_ sedimentation. All seawater parameters during the experiment are provided in Table S2. Sampling for biomarker analysis was conducted in both normal temperature and MHW groups on day 3, 10, and 13 of the MHW exposure (Fig. 1A).

### 2.3 Mortality and condition index

Mortality in each experimental group was recorded daily. Mussels exhibiting relaxed adductor muscles and fully opened valves, with no response to tactile stimulation, were classified as dead. At the designated sampling time points, soft tissues from eight mussels per treatment group were collected and dried to a constant weight at 60 °C. The condition index (CI) was calculated according to the method described by Clark et al. [54], using the following equation:

$$CI=\left(d_{\text{tm}}/d_{\text{sm}}\right)\times 100$$

where, *d*_tm_ is the dry soft tissue mass (g) and *d*_sm_ is the dry shell mass (g).

### 2.4 Determination of the number, cumulative length, and adhesion of byssal threads

A pressure gauge (DS2-50 N, ZHIQU, Shanghai, China) was employed to measure the adhesion (Text S2). Furthermore, 5 mussels that had not undergone adhesion testing were selected, and their complete byssal plaques were preserved. The byssal threads were then cut close to the foot, and the total number of byssal threads (NBT) was counted. Additionally, the length of the byssal threads was measured using a vernier caliper with an accuracy of 0.1 mm.

### 2.5 Histological observation of the mussel foot

The histopathological observation of mussels was conducted according to standard procedures. The area of the foot glands, NBG, and LCE were determined using FIJI software [55], and twelve replicates were performed for each treatment group. The severity of foot histopathology was evaluated by independent scoring two parameters [56]: the NBG and LCE. Detailed experimental protocols are explained in Text S3.

### 2.6 Measurement of the gene expression in the foot

After 3, 10, and 13 days of the corresponding treatments, six individuals were randomly selected from the experimental groups for gene expression analysis. In this study, the mRNA expression levels of fifteen genes, *Mfp-1/2/3/4/5/6* from the foot adhesion proteins, *preCOL-D/P/NG* from the pre-collagen proteins, *HSP-70/90* from the heat shock protein, and *Caspase-3*, *TLR-4*, *Myd88*, and *TNF-α* from immune and cytotoxicity-related pathways, were analyzed in the foot of mussels (qRT–PCR primers are shown in Table S3). Total RNA extraction, cDNA synthesis, and quantitative real-time PCR methods are described in Text S4.

### 2.7 Analysis of energy-related, oxidative, and immune-related parameters in the foot

Foot muscle tissue was homogenized in normal saline (1:9, w:v), centrifuged, and the supernatant was used to assess energy-related parameters, oxidative stress biomarkers, and immune parameters (Text S5). Glucose and lipid (triglyceride) reserves in the foot muscle homogenates were assessed using commercial kits (Glu: A154-1; TG: A110-1). Albumin content was assessed using an albumin detection kit (ALB, A028-2). Oxidative stress biomarkers, including activities of superoxide dismutase (SOD; A001-3), catalase (CAT; A007-1-1), and glutathione peroxidase (GPX; A005-1), as well as concentrations of lipid peroxidation products (LPO; A106-1), were also determined. Immune-related enzyme activities, acid phosphatase (ACP; A060-2), and alkaline phosphatase (AKP; A059-2), were evaluated similarly. All assays were performed according to the manufacturer’s instructions using a microplate reader (Flexstation®3, Molecular Devices, CA, USA). All enzyme assays were conducted at 28 °C. Kits were supplied by Nanjing Jiancheng Bioengineering Institute, China. Detailed protocols are provided in Text S5.

### 2.8 Analysis of cell viability and reactive oxygen species levels

Foot cells were isolated from mussels following established protocols with minor modifications (Text S6). CV and ROS were assessed using commercial kits. CA was evaluated by flow cytometry on a BD Accuri® C6 system (USA) using the Annexin V-FITC apoptosis detection kit (Beyotime Biotechnology, China).

### 2.9 Statistical analysis

The Shapiro–Wilk test and Levene’s test were used to assess data normality and homogeneity of variance, respectively. Effects of nano-TiO_2_ concentration, temperature regime, exposure time, and their interactions were analyzed by three-way analysis of variance (ANOVA). Student’s *t*-test was applied to compare differences between normal temperature and MHW at the same nano-TiO_2_ concentration and exposure time. Statistical significance was set at *P* < 0.05. Pearson correlation coefficients were calculated to assess relationships among measured parameters. These analyses were conducted using SPSS 26.0 (IBM Corp., Armonk, NY, USA). Principal component analysis (PCA) was performed using R (Rx64.0.5; rstudio.com, USA), and variable biplots were generated with the factoextra package as implemented in R.

## 3 Results

### 3.1 Characterization of nano-TiO_2_

The electron microscopy analyses showed that anatase nano-TiO_2_ particles aggregated into irregular shapes (Fig. 1B) and clustered in seawater (Fig. 1C). The average particle size of nano-TiO_2_ was 25.8 ± 2.1 nm, consistent with the manufacturer’s specifications. As the temperature increased from 22 °C to 28 °C, the hydrodynamic diameter increased from 1254 ± 64.78 nm to 2458 ± 180.84 nm, and the zeta potential decreased from 13.8 ± 1.37 mV to 6.21 ± 0.89 mV (Fig. 1D and E).

### 3.2 Impacts of MHW and nano-TiO_2_ on the survival rate and condition index

Survival was >90% in all experimental groups, and no significant effect of exposure conditions on mussel mortality was observed (*P* > 0.05; Fig. S1A). MHW had no significant effect on the CI of mussels (Fig. S1B). In mussels exposed to LT-MHW, the CI was significantly reduced (0.056 ± 0.007) compared with both the LT (0.069 ± 0.005) and the CK groups (0.074 ± 0.010) (*P* < 0.05).

### 3.3 Impacts of MHW and nano-TiO_2_ on the characteristics of the byssal threads

Three-way ANOVA showed significant effects of temperature, nano-TiO_2_ concentration, exposure time, and their interactions on NBT, adhesion of total byssal threads (ATBT), and adhesion of single byssal threads (ASBT) (*P* < 0.05; Table S4), with the only exception of the interaction of temperature × time × nano-TiO_2_, which had no significant effect on ASBT (*P* > 0.05; Table S4). Cumulative length of the byssal threads was significantly affected by exposure time, but not by temperature, nano-TiO_2_, or their interactions (Table S4).

**Fig. 2:**
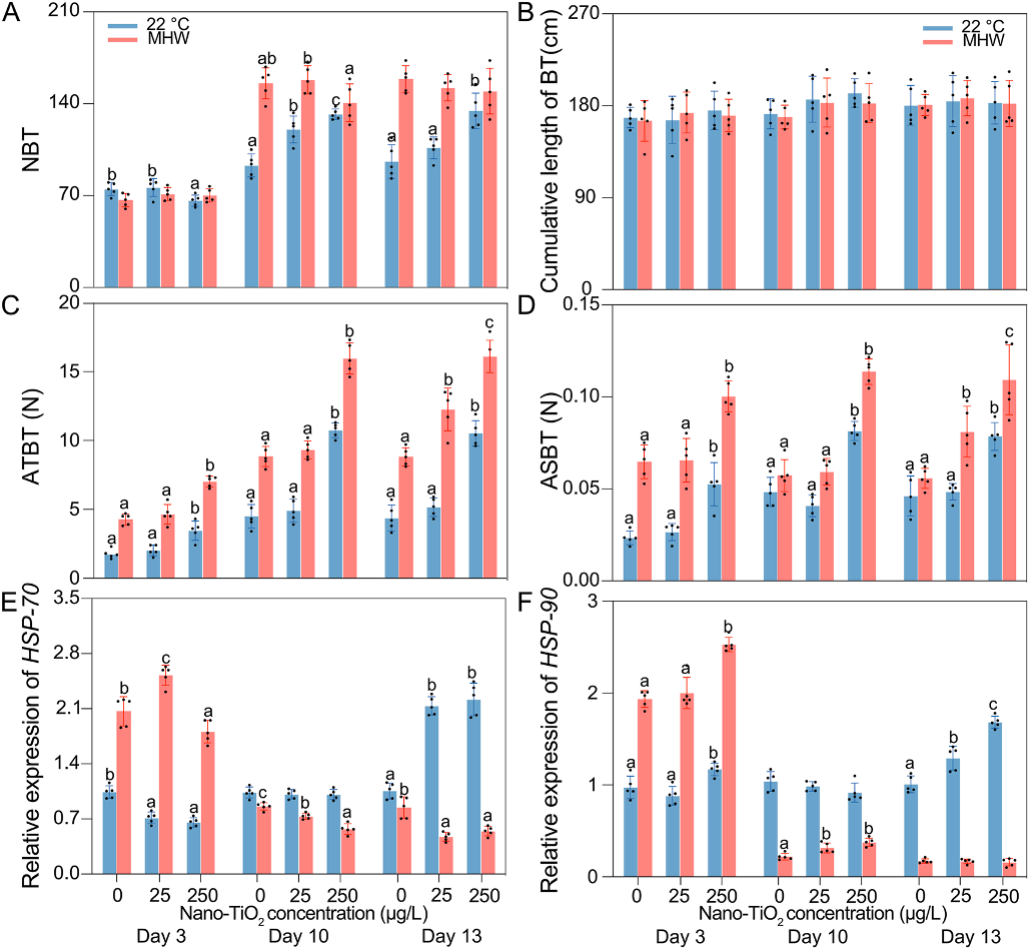
Effects of nano-TiO_2_ concentrations and marine heatwave (MHW) on (A−D) byssal thread properties, and (E) *HSP-70* and (F) *HSP-90* mRNA expression in *M. coruscus* following 3, 10, and 13 days of exposure. Different lowercase letters indicate significant differences among nano-TiO_2_ treatments (0, 25, and 250 μg/L) (*P* < 0.05). NBT, number of byssal threads; ATBT, adhesion of total byssal threads; ASBT, adhesion of single byssal threads; HSP, heat shock proteins.

On day 3, MHW exposure (66.6 ± 5.1) and HT exposure (66.2 ± 4.3) significantly reduced NBT compared to the CK (75 ± 4.8) (*P* < 0.05; Fig. 2A). NBT increased over time in all exposure groups, with the greatest increase observed under the MHW scenario. On day 10 and 13, NBT was higher under MHW conditions compared to at 22 °C, with significant increases observed in the MHW and LT-MWH groups (*P* < 0.01). At 22 °C, nano-TiO_2_ exposure led to a concentration-dependent increase in NBT after 10 days (*P* < 0.01).

The ATBT increased over time and was enhanced by both MHW and nano-TiO_2_ exposures (*P* < 0.05; Fig. 2C). HT and HT-MHW led to an increase in ATBT at all exposure times. After 13 days of exposure, a positive effect of nano-TiO_2_ on ATBT was also observed under the LT-MHW exposure (*P* < 0.05). Similar enhancing effects of MHW and nano-TiO_2_ exposure were observed for the ASBT (Fig. 2D), although the effects of MHW were less consistent across exposure times compared to those observed for ATBT.

### 3.4 Impacts of MHW and nano-TiO_2_ on histopathology of the foot

**Fig. 3:**
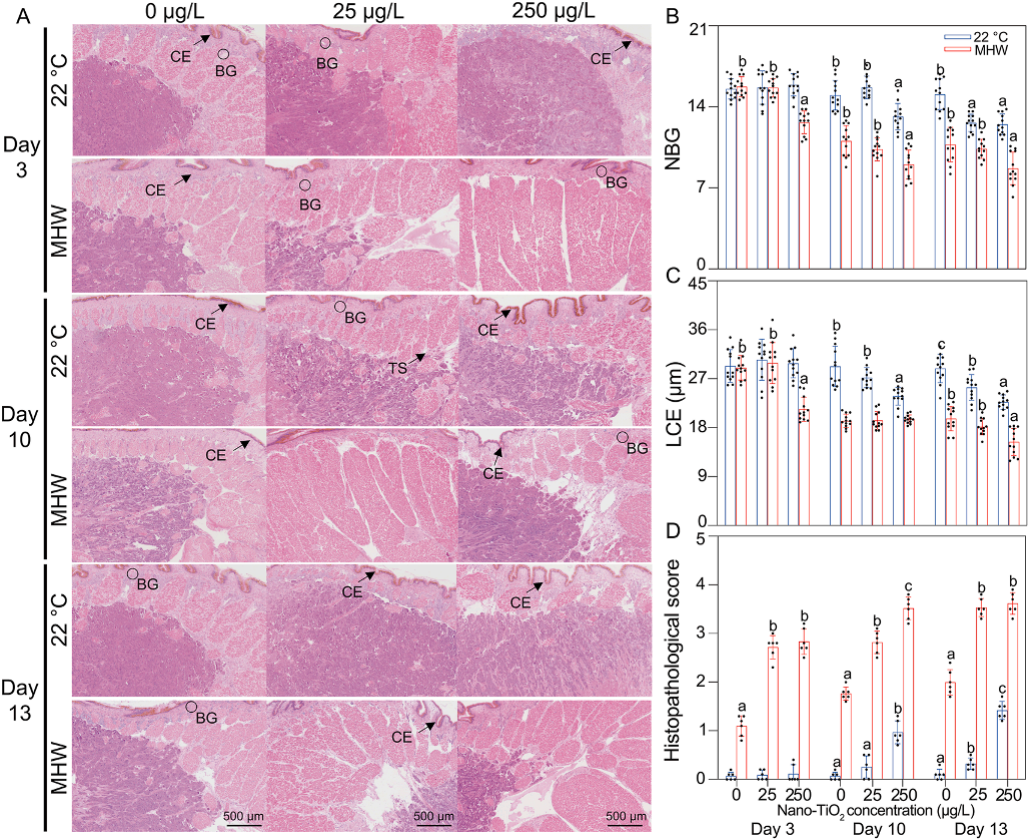
Foot tissue stained with hematoxylin and eosin (H&E) (A), number of basophilic granulocytes (NBG; B), length of the ciliated epithelium (LCE; C), and histopathological score (D) in *M. coruscus* exposed to nano-TiO_2_ (0, 25, and 250 μg/L) and different temperature conditions (22 °C and MHW) after 3-day, 10-day, and 13-day exposures. Different lowercase letters indicate significant differences among nano-TiO_2_ treatments (0, 25, and 250 μg/L) (*P* < 0.05). BG, basophilic granulocytes; CE, ciliated epithelium.

The NBG and LCE in the foot of mussels were significantly reduced following exposure to the MHW scenario (Fig. 3A–C). After 3 days of exposure, a significant decrease in NBG and LCE was observed only in mussels exposed to HT-MHW. However, at later time points (day 10 and 13), MHW-induced reductions in NBG and LCE were evident in the MHW group as well as in both nano-TiO_2_ exposure groups. Regarding LCE, significant declines under HT-MHW exposure were detected at day 3 (21.4 ± 2.2 μm), in the HT group at day 10 (23.8 ± 1.6 μm), and under both temperature regimes at day 13 (22.7 ± 1.3 μm and 15.4 ± 2.5 μm) (*P* < 0.05; Fig. 3C).

The histopathological score (HS) of the foot tissue was significantly elevated in MHW-exposed mussels, irrespective of nano-TiO_2_ concentration or exposure duration. Additionally, exposure to LT-MHW and HT-MHW further increased the HS after 10 and 13 days of exposure (Fig. 3D). In mussels maintained at the normal temperature (22 °C), the effects of nano-TiO_2_ on HS were less pronounced, with significant increases observed at 250 μg/L after 10 and 13 days, and at 25 μg/L only after 13 days of exposure.

At day 13, the relative foot gland area was reduced under HT and HT-MHW conditions by 17.5% and 46.7%, respectively (*P* < 0.05), compared to the CK group (Fig. S2A–G). In addition, MHW exposure significantly decreased the percentage of foot gland area irrespective of nano-TiO_2_ treatment (Fig. S2G).

### 3.5 Expression of the heat shock proteins in the foot tissue

Temperature, nano-TiO_2_ concentration, exposure time, and their interactions had significant effects on the transcript levels of *HSP-70* and *HSP-90* in the foot tissue (*P* < 0.05; Table S4), except for the time × nano-TiO_2_ concentration interaction on *HSP-90* (*P* > 0.05).

On day 3, MHW exposure significantly increased the mRNA expression of *HSP-70* and *HSP-90* compared to the CK group (*P* < 0.05; Fig. 2E and F). Over time, *HSP-70* and *HSP-90* mRNA levels decreased in MHW-exposed mussels, regardless of nano-TiO_2_ exposure. Prolonged exposure (13 days) to LT or HT led to significant overexpression of both *HSP-70* and *HSP-90* mRNA (*P* < 0.05).

### 3.6 Impacts of MHW and nano-TiO_2_ on mRNA expression of byssus-related proteins

Temperature, nano-TiO_2_ concentration, exposure time, and their interactions had significant effects on the mRNA expression of all studied byssus-related genes (*Mfp-1*, *Mfp-2*, *Mfp-3*, *Mfp-4*, *Mfp-5*, *Mfp-6*, *preCOL-D*, *preCOL-P*, and *preCOL-NG*) (*P* < 0.05; Table S5).

**Fig. 4:**
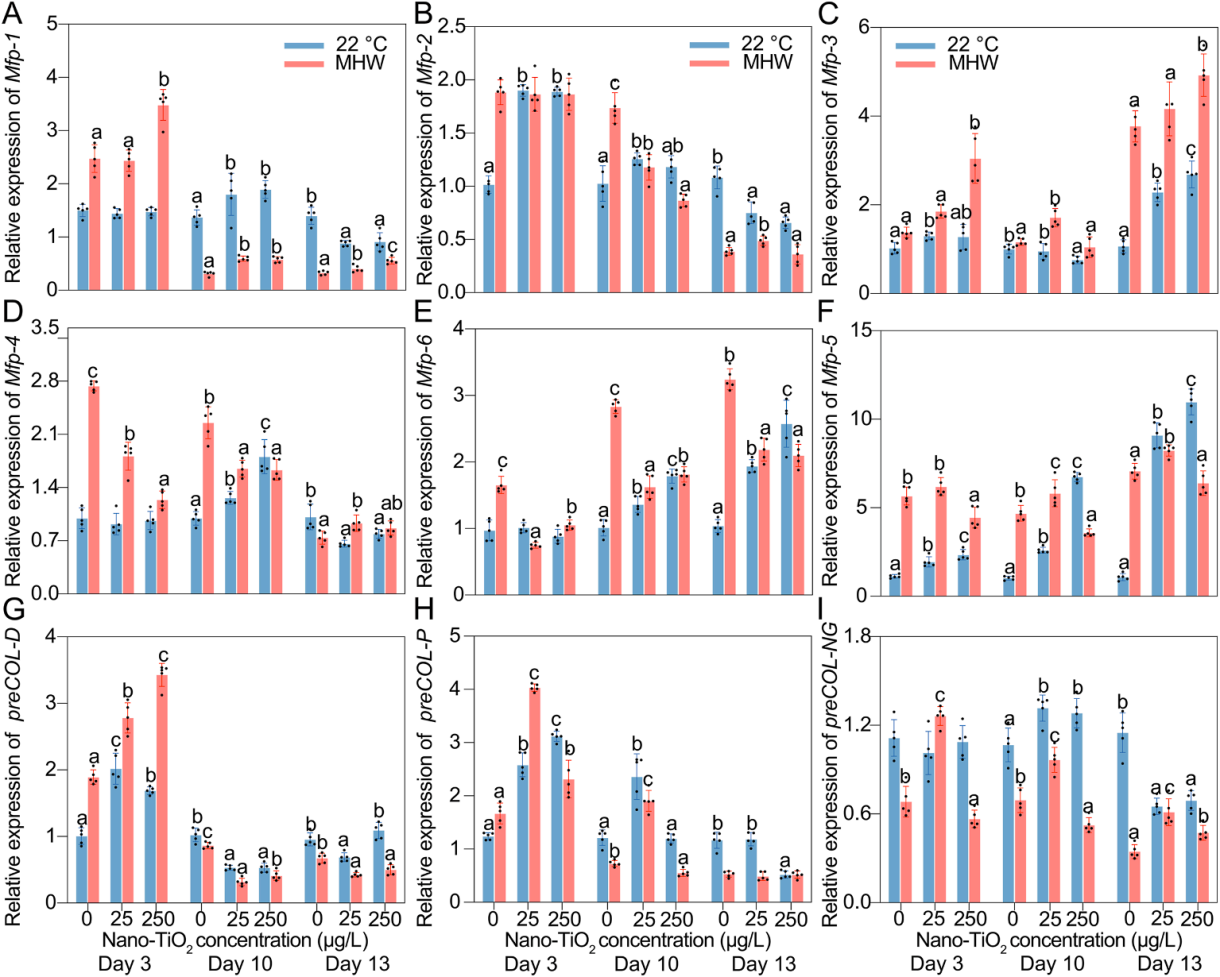
Effects of nano-TiO_2_ (0, 25, and 250 μg/L) and temperature regime (22 °C and MHW) on the mRNA expression of byssus-related proteins in the foot of *M. coruscus* after 3-day, 10-day, and 13-day exposures. (A) *Mfp-1,* (B) *Mfp-2*, (C) *Mfp-3*, (D) *Mfp-4,* (E) *Mfp-5,* (F) *Mfp-6,* (G) *preCOL-D*, (H) *preCOL-P*, and (I) *preCOL-NG*. Different lowercase letters indicate significant differences among 0, 25, and 250 μg/L nano-TiO_2_ treatments (*P* < 0.05).

On day 3, LT-MHW and HT-MHW exposure significantly upregulated the relative expression of *Mfp-1*, *Mfp-3*, *Mfp-4*, *Mfp-5*, and *preCOL-D* (*P* < 0.05; Fig. 4A,C-E,G). After 10 and 13 days of exposure, MHW significantly suppressed the mRNA expression of *Mfp-1*. Exposure to 250 μg/L nano-TiO_2_ upregulated *Mfp-*1 mRNA levels in the mussel foot, with significant increases observed in the HT-MHW group at day 3 and 13, and in HT and HT-MHW regimes at day 10 (*P* < 0.05; Fig. 4A).

The transcript levels of *Mfp-4* were enhanced by MHW exposure across all nano-TiO_2_ treatments at day 3 and 10 (*P* < 0.05; Fig. 4D). Increased *Mfp-3* expression was observed in response to HT and HT-MHW regimes at day 3 and 13, but not at day 10 (Fig. 4C). At day 3 and 10, MHW exposure upregulated *Mfp-2* expression, whereas at day 10, it downregulated *Mfp-2* expression in the HT-MHW group. By day 13, MHW, LT-MHW, and HT-MHW exposure suppressed *Mfp-2* transcript levels (*P* < 0.05; Fig. 4B). LT and HT exposure enhanced *Mfp-*2 mRNA levels at day 3 and 10 but suppressed them at day 13.

At day 10, the mRNA expression levels of *Mfp-5* and *Mfp-6* were upregulated by MHW and LT-MHW groups; however, this enhancing effect was negated by co-exposure to the HT-MHW (Fig. 4E and F). At day 13, the MHW-induced upregulation of mRNA expression was observed only in the MHW group for *Mfp-5* and *Mfp-6*, and in the LT-MHW group for *Mfp-4*. LT and HT exposure enhanced the mRNA expression levels of *Mfp-5* and *Mfp-6* (*P* < 0.05; Fig. 4D−F). At 22 °C, transcript levels increased in a concentration-dependent manner with rising nano-TiO_2_ concentration: for *Mfp-5* at day 3, for *Mfp-4*, *Mfp-5*, and *Mfp-6* at day 10, and for *Mfp-5* and *Mfp-6* at day 13 (*P* < 0.05; Fig. 4D−F).

Following an early (day 3) upregulation (1.9 ± 0.11), MHW exposure significantly suppressed the mRNA expression of *preCOL-D* after 10 (0.86 ± 0.05) and 13 (0.67 ± 0.07) days of exposure (*P* < 0.05; Fig. 4G). In the LT and HT groups, *preCOL-D* levels were elevated to 2-fold and 1.7-fold of the respective controls at day 3 but were significantly suppressed by about 48.1% and 48.2%, respectively, at day 10 compared to the corresponding control groups. A similar nano-TiO_2_-induced suppression trend was observed after 13 days of exposure, although this was only significant in the MHW-exposed mussels.

The mRNA levels of *preCOL-P* were initially elevated in response to MHW and LT-MHW exposure at day 3, but were suppressed on day 10 in all nano-TiO_2_-exposed groups (*P* < 0.05; Fig. 4H). At day 3 in the 22 °C group, *preCOL-P* transcription levels increased in a concentration-dependent manner to 2.1-fold and 2.5-fold of the control. After 13 days of exposure, *preCOL-P* mRNA levels decreased in response to HT, LT-MHW, and HT-MHW.

The expression of *preCOL-NG* was generally suppressed by MHW exposure at all time points, except for the 25 μg/L nano-TiO_2_ group (*P* < 0.05; Fig. 4I). The effects of nano-TiO_2_ on *preCOL-NG* mRNA expression varied across temperature regimes and time points. In mussels kept at 22 °C, *preCOL-NG* mRNA levels were elevated in both nano-TiO_2_-exposed groups compared to the control at day 10, but expression was reduced in response to nano-TiO_2_ exposure at day 13 (*P* < 0.05).

### 3.7 Impacts of MHW and nano-TiO_2_ on bioenergetics-related parameters in the foot muscle

The concentrations of Glu, TG, and ALB in the mussels’ foot were significantly affected by temperature, nano-TiO_2_ concentration, exposure time, and their interactions (*P* < 0.01; Table S6).

**Fig. 5:**
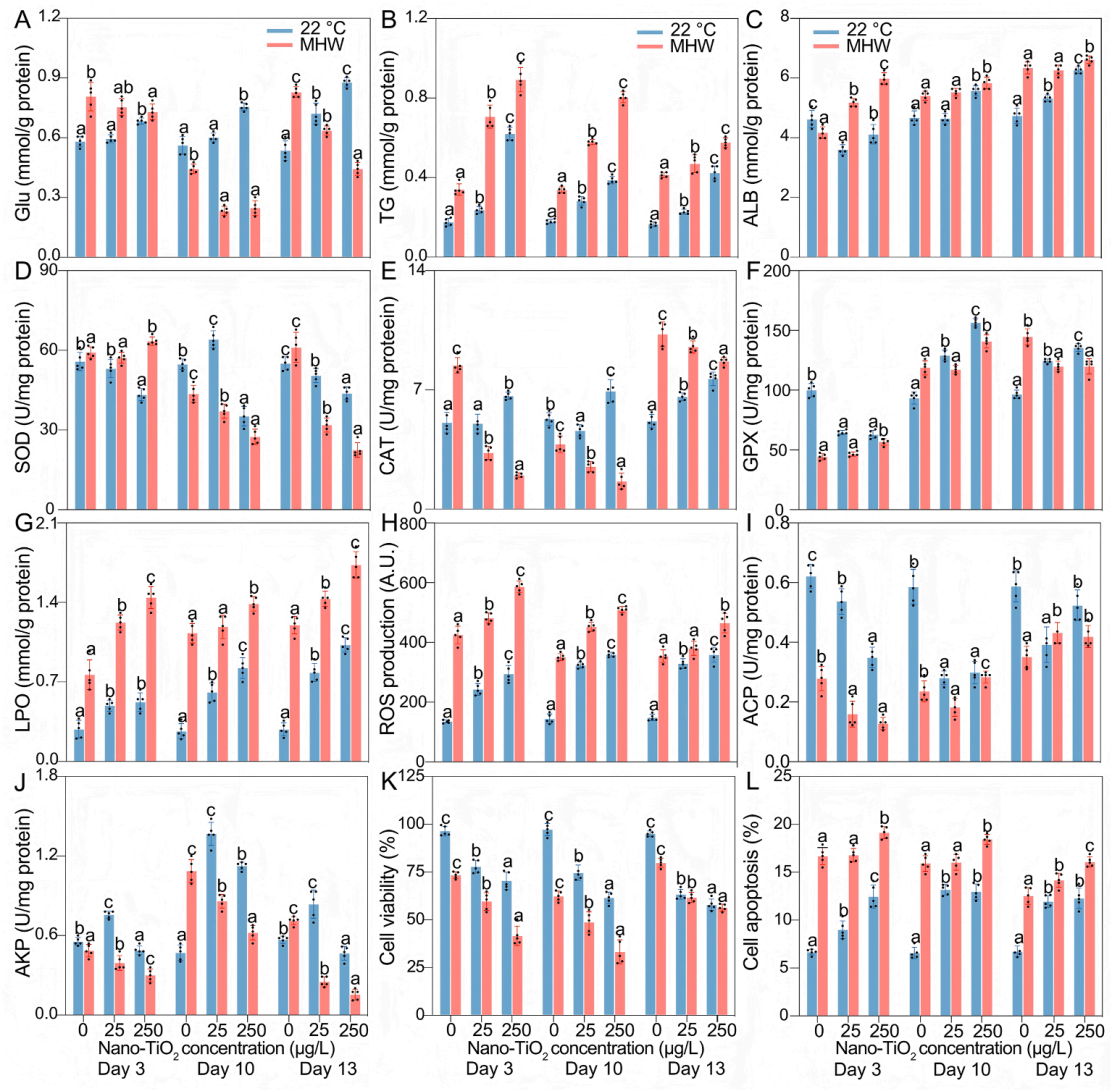
Effects of nano-TiO_2_ (0, 25, and 250 μg/L) and temperature regime (22 °C and MHW) on energy, redox, immune responses, and cell viability-related parameters in the foot of *M. coruscus* after 3-day, 10-day, and 13-day exposures. (A) Glu, (B) TG, (C) ALB, (D) SOD, (E) CAT, (F) GPX, (G) LPO, (H) ROS production, (I) ACP, (J) AKP, (K) cell viability, and (L) cell apoptosis. Lowercase letters indicate significant differences between 0, 25, and 250 μg/L nano-TiO_2_ treatments (*P* < 0.05). ACP, acid phosphatase; AKP, alkaline phosphatase; ALB, albumin; CAT, catalase; Glu, glucose; GPX, glutathione peroxidase; LPO, lipid peroxidation; ROS, reactive oxygen species; SOD, superoxide dismutase; TG, triglycerides.

During the warming phase (day 3), MHW-exposed mussels showed higher Glu levels (0.81 ± 0.07 mmol/g protein) in the foot compared to the CK group (0.58 ± 0.03 mmol/g protein) (*P* < 0.05; Fig. 5A). However, by the end of the heatwave (day 10), Glu levels in the MHW-exposed mussels significantly decreased to 23.6% of those in mussels kept at 22 °C. At 22 °C, nano-TiO_2_ exposure resulted in a concentration-dependent increase in Glu content across at day 10 (*P* < 0.05). In contrast, in MHW-exposed mussels, a concentration-dependent decrease in Glu levels was observed as nano-TiO_2_ concentration increased at day 13 (*P* < 0.05).

The foot muscle levels of TG increased in response to the MHW scenario compared to the normal temperature, as well as in response to increasing nano-TiO_2_ concentrations under both normal temperature and MHW conditions, across all exposure times (*P* < 0.05; Fig. 5B).

Exposure to LT and HT resulted in a decrease to 22.1% (25 μg/L) and 11.1% (250 μg/L) in ALB content of the CK level during the early exposure phase (day 3), but led to a concentration-dependent increase in ALB content at day 13 of exposure. In MHW-exposed mussels, nano-TiO_2_ exposure caused a concentration-dependent increase in ALB content in the foot at day 3 (*P* < 0.05; Fig. 5C).

### 3.8 Impacts of MHW and nano-TiO_2_ on oxidative stress parameters

Temperature, nano-TiO_2_ concentration, exposure time, and their interactions had significant effects on SOD, CAT, GPX, LPO, and cellular ROS levels (*P* < 0.01; Table S6).

In general, exposure to increasing concentrations of nano-TiO_2_ led to a decrease in SOD activity in the mussel foot across all exposure times, under both normal temperature and MHW conditions (*P* < 0.05; Fig. 5D). The only exception was observed in MHW-exposed mussels on day 3, where exposure to HT-MHW resulted in an increase in SOD activity.

Exposure to the MHW resulted in elevated CAT activity on day 3, as well as in the MHW, LT-MHW, and HT-MHW groups on day 13 (*P* < 0.05; Fig. 5E). On day 10 (the end of the heatwave), MHW-exposed mussels showed lower CAT activity compared to their counterparts kept at 22 °C, regardless of nano-TiO_2_ exposure conditions. Exposure to HT led to enhanced CAT activity at all time points. In MHW-exposed mussels, CAT activity declined with increasing nano-TiO_2_ concentrations at all time points (*P* < 0.05; Fig. 5E).

At 22 °C, exposure to nano-TiO_2_ led to a decrease to 35.4% (LT) and 37% (HT) in GPX activity on day 3, but a concentration-dependent increase on day 10 and 13 (*P* < 0.05; Fig. 5F). Exposure to HT-MHW resulted in elevated GPX activity on day 3 (56.7 ± 2.7 U/mg protein) and 10 (140.9 ± 5.6 U/mg protein), but decreased GPX activity on day 13 (120.0 ± 6.4 U/mg protein), compared with the respective MHW controls (*P* < 0.05; Fig. 5F).

LPO content in the mussel foot was consistently higher under the MHW scenario compared to the CK (*P* < 0.05; Fig. 5G). Exposure to nano-TiO_2_ led to a concentration-dependent increase in LPO content at both temperature regimes, with the highest levels observed by day 13 in mussels exposed to HT-MHW (*P* < 0.05).

ROS production was elevated by MHW exposure across all time points (*P* < 0.05; Fig. 5H). Exposure to nano-TiO_2_ resulted in a concentration-dependent increase in cellular ROS production under both temperature regimes. The highest ROS levels (586 ± 19.6 A.U.) were recorded on day 3 in mussels exposed to HT-MHW (*P* < 0.05).

### 3.9 Impacts of MHW and nano-TiO_2_ on immune-related parameters

Enzyme activities of ACP and AKP, as well as mRNA expression of *TLR-4* and *Myd88,* were significantly affected by temperature, nano-TiO_2_ concentration, exposure time, and their interactions (*P* < 0.01; Tables S6 and S7). The only exception was the time × nano-TiO_2_ interaction, which showed no significant effect on *Myd88* expression (*P* > 0.05; Table S7).

**Fig. 6:**
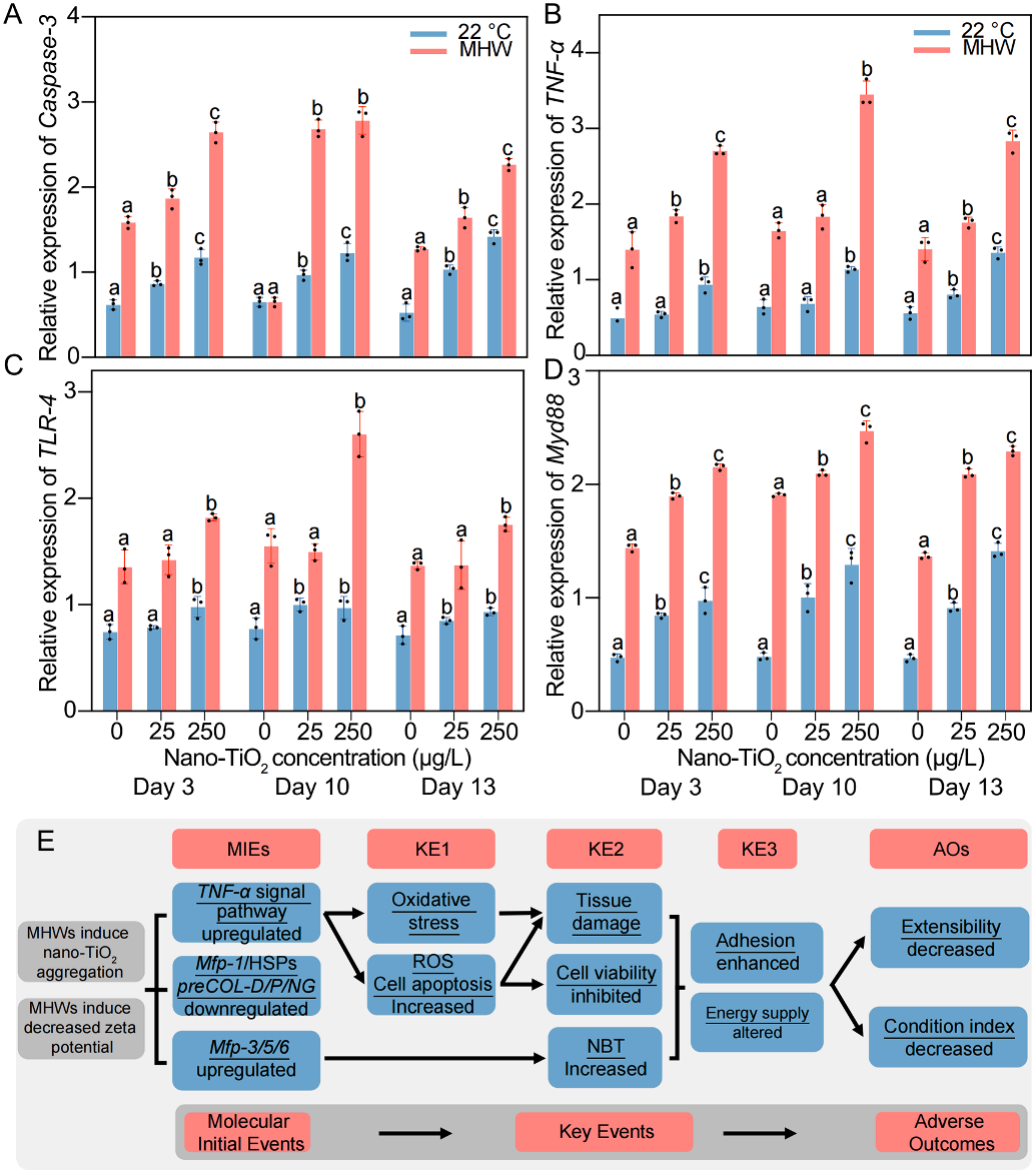
Effects of nano-TiO_2_ (0, 25, and 250 μg/L) and temperature regime (22 °C and MHW) on the mRNA expression of immune- and cytotoxicity-related genes in the foot of *M. coruscus* after 3-day, 10-day, and 13-day exposures. (A) *Caspase-3*, (B) *TNF-α*, (C) *TLR-4*, and (D) *Myd88*. Lowercase letters indicate significant differences among 0, 25, and 250 μg/L nano-TiO_2_ treatments (*P* < 0.05). (E) A predicted adverse outcome pathways (AOPs) network of MHW and nano-TiO_2_ with mussel model.

MHW treatment significantly inhibited ACP and AKP activities on day 3 compared with the CK group (*P* < 0.05; Fig. 5I and J). LT and HT treatment resulted in a significant decrease in ACP activities at day 3 (*P* < 0.05; Fig. 5I). On day 10, MHW significantly decreased ACP activities and increased AKP activities in the MHW group and the LT-MHW compared with the CK group. Under MHW, AKP activity was significantly inhibited by nano-TiO_2_ exposure in a concentration-dependent manner. Under MHW, ACP activity was increased by nano-TiO_2_, but AKP activity was inhibited in a concentration-dependent manner at day 13 (*P* < 0.05; Fig. 5I and J). Both nano-TiO_2_ exposure and MHW exposure significantly upregulated the relative expression of *TLR-4* and *Myd88* compared with the control group (*P* < 0.05; Fig. 6C and D). Besides, day 10-exposure significantly upregulated the expression of *TLR-4* and *Myd88* compared to day 3 and day 13 in the HT-MHW treatments (*P* < 0.05).

### 3.10 Impacts of MHW and nano-TiO_2_ on cytotoxicity-related traits

Three-way ANOVA illustrated that temperature, nano-TiO_2_ concentration, exposure time, and their interactions had significant effects on CV, CA, ROS, *Caspase-3*, and *TNF-α* (*P* < 0.01; Tables S6 and S7).

On day 3, 10 and 13, MHW significantly reduced CV compared with the control group by 23.9%, 35.8%, and 15.9%, respectively. At both normal temperature and MHW, nano-TiO_2_ exposure resulted in a significant reduction in CV and showed a significant concentration dependence (*P* < 0.05; Fig. 5K). CV decreased first (day 10) and then significantly increased (day 13) under MHW treatments (*P* < 0.05).

At day 3, 10, and 13, MHW significantly increased CA to 2.5-, 2.4-, and 1.9-fold of the control, respectively. Exposure to HT and HT-MHW resulted in a significant increase in CA on day 3, 10, and 13, with a significant concentration dependence in the normal temperature treatments on day 3 and the MHW treatments on day 13 (*P* < 0.05; Fig. 5L). Compared with day 3 (16.7% ± 0.9%) and day 10 (15.9% ± 0.8%), CA decreased significantly in the MHW treatments on day 13 (12.5% ± 0.8%).

Both nano-TiO_2_ exposure and MHW exposure significantly upregulated the relative expression of *Caspase-3* and *TNF-α* compared with the control group (*P* < 0.05; Fig. 6A and B).

### 3.11 Pearson coefficient analysis and PCA

Pearson coefficient analysis showed that the ATBT was positively correlated with NBT, *Mfp-3*, *Mfp-5*, and *Mfp-6* (*P* < 0.01; Fig. S3A–D). LPO content was negatively correlated with CV, ACP, AKP, and SOD (*P* < 0.01) and significantly positively correlated with CA, ROS, TG, and ALB (*P* < 0.05; Fig. S4).

The first two principal components of PCA explained 66.5% of the total variance, with PC1 accounting for 47.5% and distinguishing between the day 3 treatments and those on days 10 and 13. PC2 showed clustering of samples within the exposed groups (Fig. S5A). For the physiological and biochemical indices, the first two principal components of PCA explain 64.3% of the variance (Fig. S5B). PC1 (46.6%) separated the normal temperature treatments from the MHW treatments, while PC2 (17.7%) distinguished the day 3 treatments from the day 13 treatments.

## 4 Discussion

Byssus production is vital for mussels, enabling firm attachment to substrates in dynamic aquatic environments and facilitating the formation of mussel beds that enhance habitat complexity and biodiversity. Despite its ecological importance, byssus synthesis incurs high energetic costs due to the production of complex proteins and polyphenolic compounds [19,57]. Mussels often prioritize byssus formation over growth and reproduction, particularly under high hydrodynamic stress, as insufficient anchorage can result in dislodgement and mortality [19,57]. Our study indicated that nano-TiO_2_ pollution and MHW adversely affected the health of the mussel foot, the primary organ responsible for byssus production. However, we observed complex effects on byssus production and performance, suggesting possible compensatory mechanisms that prioritize investment in byssus synthesis due to its critical ecological importance.

### 4.1 Oxidative stress as an early key event triggered by MHW and nano-TiO_2_ exposure

Oxidative stress is a hallmark of the generalized stress response in marine organisms. It is commonly triggered by various environmental stressors, including pollutants and heat [58], and is therefore widely regarded as an effective and sensitive KE for assessing organismal responses to both chemical and thermal stressors. Our study demonstrated that exposure to both MHW and nano-TiO_2_ increased ROS production in mussels, resulting in an imbalance of antioxidant defenses and a significant elevation in LPO, a well-established marker of oxidative damage. These findings are consistent with known stress mechanisms of nano-TiO_2_ and elevated temperatures, both of which commonly induce excessive ROS production and disrupt cellular redox homeostasis in mussels [58,59]. In the present study, we observed that exposure to LT-MHW and HT-MHW conditions initially suppressed the activities of antioxidants CAT and GPX in mussels. However, this suppression was not sustained and was followed by a recovery phase (after 10-d exposure), with increased antioxidant activity later in the exposure period. In contrast, SOD activity was persistently suppressed throughout the exposure. These findings suggest that while some antioxidant defenses may recover over time, the overall oxidative protection could be dysregulated due to an imbalance between the activities of SOD and CAT, which may impair the cell’s ability to effectively manage oxidative stress [60,61]. The accumulation of LPO products under both single and combined nano-TiO_2_ and MHW exposures supports this conclusion and indicates ongoing oxidative stress.

### 4.2 Heat shock response under thermal and nanoparticle stress

HSPs are synthesized in response to stressors such as heat, oxidative stress, and pollutants. They function as molecular chaperones, refolding damaged proteins and tagging irreparably damaged ones for degradation [62,63]. The early upregulation of mRNA levels of *HSP-70* and *HSP-90* during the MHW exposure of *M. coruscus* likely reflects a compensatory response to stress-induced damage in the cellular proteome [64,65]. However, by day 10 and 13, we observed a decline in the expression of these chaperones. The decline on day 13 could be attributed to a recovery phase, as the temperature returned to baseline levels (22 °C). However, the decrease in HSP expression on day 10, which coincided with the seventh day of MHW exposure, is more intriguing. This suggests that after prolonged exposure, the capacity for HSP synthesis may be overwhelmed or less effective. Similar trends have been observed in marine bivalves, where a decline in HSP expression during extended heat exposure has been interpreted as a shift away from HSP-mediated protection in favor of other, potentially more sustainable, protective mechanisms that emerge during thermal acclimation [65,66]. In contrast to the MHW, exposure to nano-TiO_2_ resulted in elevated mRNA levels of *HSP-70* and *HSP-90* only after prolonged (day 13) exposure, indicating that this stressor induces a slower accumulation of misfolded proteins. This gradual buildup of misfolded proteins seems to stimulate chaperone expression more progressively compared to the immediate and acute effects observed during warming.

### 4.3 Activation of apoptotic and inflammatory pathways following cellular stress and altered immune enzyme activities

Exposure to LT-MHW and HT-MHW significantly elevated the expression of *Caspase-3, TLR-4, Myd88,* and *TNF-α* mRNA in the mussels. Activation of the *TLR-*4/NF*-κB* signaling pathway promotes inflammation by upregulating pro-inflammatory cytokines such as *Myd88* and *TNF-α* [67], whereas the caspase family of proteases plays a crucial role in apoptosis, a programmed process that removes damaged cells and helps mitigate inflammation [68]. The observed decrease in *HSP-70* and *HSP-90* mRNA expression, coupled with increased levels of *Caspase-*3 mRNA and activation of the *TLR-*4/NF*-κB* pathway, suggests that the proteome damage caused by heat and/or oxidative stress may act as a key initiating event (MIE) driving apoptosis and inflammation in foot tissue [[69], [70], [71]]. The alterations in apoptosis- and inflammation-related molecular pathways at the cellular level were reflected in downstream increased apoptosis, reduced cell viability, and heightened histopathological damage in the mussel foot during simulated MHW and nano-TiO_2_ exposures. These findings suggest that oxidative stress and proteome damage surpass the capacity of the cellular protective mechanisms, leading to cellular dysfunction and tissue damage. Interestingly, the cytotoxic effects of MHW were rapidly evident, appearing as early as day 3 of exposure, and persisted throughout the MHW and recovery period. In contrast, nano-TiO_2_ exposure led to a more gradual progression of cytotoxicity and tissue damage over time. Previous studies have also shown that exposure to pollutants or high temperatures can modulate the expression of apoptotic and inflammation-related genes [65,72,73], inducing programmed cell death and ultimately histopathological damage [[74], [75], [76]]. Within the AOP framework, we propose that increased apoptosis and inflammation, along with reduced cell viability, may underlie the adverse tissue-level outcomes, as shown by the severe damage observed in the mussels’ foot (Fig. 6E).

Alterations of apoptosis and inflammation pathways in the foot muscle of *M. coruscus* exposed to MHW and nano-TiO_2_ was linked to changes in two key immune-related enzymes: AKP and ACP. AKP plays critical tissue-specific roles in regulating immune responses, apoptosis, and inflammation, and has been shown to facilitate the removal of apoptotic cells while also mitigating inflammatory processes [77,78]. ACP, on the other hand, may indirectly contribute to these pathways, potentially through processes like autophagy or lysosomal degradation of cellular components damaged by stressors [79,80]. In *M. coruscus*, both AKP and ACP activities were suppressed under combined MHW and nano-TiO_2_ exposures during the early warming phase (3 days), indicating a suppression of the foot’s protective immune response. Notably, ACP activity was also reduced under MHW alone during this initial phase, even in the absence of nano-TiO_2_. AKP activity increased under single nano-TiO_2_ exposure but decreased under combined MHW and nano-TiO_2_ exposure, suggesting higher sensitivity to the nanoparticle stressor. These findings suggest that high temperature and nano-TiO_2_ stress may exert divergent effects on different enzymatic components of the immune system in marine bivalves, potentially leading to an imbalance in immune defense. Given the putative roles of AKP and ACP in apoptotic cell clearance and inflammation mitigation, suppression of these enzyme activities at different stages of MHW progression may lead to the accumulation of apoptotic cells and contribute to tissue damage in mussels under heat stress. In contrast, nano-TiO_2_ appeared to upregulate AKP and ACP activities, which may help delay the onset of apoptosis and tissue damage under control temperatures.

### 4.4 Compensatory enhancement of byssus production and adhesion under environmental stress

Remarkably, titanium concentrations in mussel foot tissues were below the limit of detection under all exposure conditions, and no measurable bioaccumulation attributable to particle internalization was detected. Besides, despite the negative effects of MHW and nano-TiO_2_ on cellular and tissue integrity in the foot (the main byssus-producing organ), the number and adhesion strength of byssal threads increased under HT-MHW in *M. coruscus*. Previous studies have shown that acute exposure to high temperature (31 °C) can reduce both the number and mechanical strength of byssal threads, whereas short-term moderate warming does not significantly alter thread number but still impairs mechanical performance [23]. In the present study, prolonged MHW exposure (13 days) was associated with increased byssal thread production despite reduced thread extensibility, suggesting the activation of compensatory mechanisms to maintain attachment performance. One such compensatory mechanism may involve the modulation of mussel foot protein (MFP) mRNA expression, which increased during the warming phase of the MHW. This upregulation was observed across all studied MFPs (*Mfp-1, Mfp-2, Mfp-3, Mfp-4, Mfp-5*, and *Mfp*-*6*). Interestingly, nano-TiO_2_ exposure attenuated the stimulatory effects of MHW on the expression of most MFPs, with the exception of *Mfp-3, Mfp-5* and *Mfp-6*, which remained upregulated under single nano-TiO_2_ exposure. Given the important roles of mussel foot proteins (particularly *Mfp-3, Mfp-4, Mfp-5,* and *Mfp-6*) in plaque formation and maturation [[81], [82], [83], [84]], their upregulation may have contributed to the increased adhesion observed in the byssus of mussels exposed to MHW and nano-TiO_2_. This hypothesis is supported by the observed positive correlation between *Mfp-3*, *Mfp-5*, and *Mfp-*6 mRNA levels and the adhesion of total byssal threads. The transient upregulation of *Mfp-1*, which is involved in forming the protective outer coating of the byssal thread [81,82], may have supported improved structural integrity of the threads; however, this upregulation was not sustained over time. Similar to *Mfp-1*, the mRNA expression of structural preCOL (collagenous modular) proteins, which form a semi-crystalline framework within the byssal thread [81,82], was initially upregulated by MHW (*preCOL-D* and *preCOL-P*), but this upregulation was not sustained during prolonged exposure. However, the observed enhancement in byssal adhesion strength may also stem from physico-chemical changes induced by MHW or nano-TiO_2_ exposure. Such changes—including alterations in the biochemical processes of byssal thread production, assembly, and maturation or shifts in the redox conditions of the adhesive environment—could have contributed to the outcome [18,21,29]. This possibility is suggested, for instance, by a general decreasing trend in both NBG and LCE observed at the highest nano-TiO_2_ concentration. These processes were not independently quantified in the present study. Therefore, the underlying mechanisms linking environmental stressors to changes in byssal adhesion remain to be fully elucidated.

### 4.5 Energetic reallocation supports byssus maintenance under MHW and nano-TiO_2_ exposure

Simulated MHW and nano-TiO_2_ exposure had no overall adverse effects on the content of energy substrates—Glu and TG—or ALB in the foot of *M. coruscus*. Except for a transient decrease in glucose levels at day 10 in mussels exposed to MHW and at day 10 and 13 in the combined exposure group, energy-rich compounds generally increased in response to both stressors. The rise in energy substrates, particularly the highly energy-dense TG, suggests an elevated energy supply to the foot muscle, which supports the metabolic demand for byssus synthesis under heat and nanopollutant stress. Since our analysis focused solely on the foot, the primary byssus-producing organ, we cannot determine whether this increase reflects a systemic upregulation of energy storage or a targeted import of energy substrates to the foot. Although elevated energy levels might be attributed to increased food intake, this seems unlikely, as previous studies have shown that both extreme temperatures and nanoparticle exposure commonly reduce filtration rates and thus food consumption in mussels [[85], [86], [87]]. The mobilization of energy substrates to the foot aligns with reports that byssus production is energetically demanding and prioritized by mussels under stress [19,57]. Additionally, the CI indicated that neither MHW nor nano-TiO_2_ alone significantly impaired the overall energetic status. However, CI was decreased by HT-MHW, indicating reduced growth or possible biomass reabsorption under these extreme stress conditions. Further studies are needed to assess systemic changes in bioenergetics to determine whether the mobilization of energy substrates to the foot under MHW, nano-TiO_2_, or their combined exposure occurs at the expense of the whole-organism energy status or other energy-demanding functions such as growth and reproduction.

## 5 Conclusions

This study, within the framework of AOPs, revealed the toxic effects of MHW and nano-TiO_2_ on the byssal defense performance of *M. coruscus*. The results suggested that oxidative stress may serve as a key early event, triggering a cascade of downstream effects that activate inflammatory and apoptotic pathways, ultimately leading to tissue damage, impaired energy allocation, and altered physiological status at the organismal level. Although mussels were able to sustain attachment through compensatory enhancement of byssal thread production, this adaptation occurred at the expense of overall energy balance, suggesting a potential decline in long-term resilience.

Several limitations of the present study should be acknowledged. First, titanium accumulation was assessed solely in the foot tissue, limiting characterization of its systemic distribution. To better elucidate tissue-specific accumulation and systemic toxicity, future analyses should include additional target organs (e.g., gills and digestive glands). Second, the lack of time-course monitoring of nano-TiO_2_ aggregation and sedimentation during the exposure period may introduce uncertainty regarding the actual exposure concentrations. To improve the precision of future toxicological evaluations, real-time characterization of nanoparticle behavior should be incorporated. Third, future research should focus on elucidating the causal relationship between oxidative stress and energy allocation to clarify whether the compensatory enhancement of byssus production represents a sustainable adaptive mechanism or merely a short-term physiological trade-off.

## CRediT authorship contribution statement

**Shuaishuai Wei:** Data curation, Formal analysis, Investigation, Methodology, Software, Visualization, Writing – original draft. **Zhihan Tu:** Data curation, Methodology, Writing – original draft. **Inna Sokolova:** Data curation, Supervision, Writing – review & editing. **Liming Chen:** Investigation, Writing – original draft. **Shixiu Wang:** Methodology, Visualization. **Menghong Hu:** Methodology, Supervision, Writing – review & editing. **Youji Wang:** Funding acquisition, Supervision, Writing – review & editing.
