# Supplementary material for: Adverse outcome pathways of titanium dioxide nanoparticles and marine heatwaves on byssal performance in mussels
Source: Eco Environ Health. 2026 Jul 17;5(3):100265. doi: 10.1016/j.eehl.2026.100265 (PMC13475504; doi:10.1016/j.eehl.2026.100265)
Supplement: Supplementary file 1 [file mmc1.docx]

**Supporting Information for:**

**Adverse outcome pathways of titanium dioxide nanoparticles and marine heatwaves on byssal performance in mussels**

Shuaishuai Wei ^a,b^, Zhihan Tu ^a,b^, Inna Sokolova ^c,d^, Liming Chen ^a,b^, Shixiu Wang ^a,b^, Menghong Hu ^a,b,e^, Youji Wang ^a,^^b,*^

^a^ International Research Center for Marine Biosciences at Shanghai Ocean University, Ministry of Science and Technology, Shanghai Ocean University, Shanghai 201306, China

^b^ Key Laboratory of Exploration and Utilization of Aquatic Genetic Resources, Ministry of Education, Shanghai Ocean University, Shanghai 201306, China

^c^ Department of Marine Biology, Institute for Biological Sciences, University of Rostock, Rostock, Germany

^d^ Department of Maritime Systems, Interdisciplinary Faculty, University of Rostock, Rostock, Germany

^e^ Marine Biomedical Science and Technology Innovation Platform of Lin-gang Special Area, Shanghai, China

*** Corresponding author:** Youji Wang, Email: youjiwang2@gmail.com

**Summary (17 Pages, 7 Tables, 5 Figures, 6 texts)**

Text S1. Methods for detection of titanium.

Text S2. Determination of the adhesion of byssal threads.

Text S3. Methods for histological observation of the mussel foot.

Text S4. Methods for gene expression.

Text S5. Methods for energetic, oxidative, and immune-related parameters in foot.

Text S6. Methods for cytotoxicity.

Table S1. Measured working concentrations of nano-TiO_2_ used in the present study.

Table S2. Seawater parameters were measured in the present study.

Table S3. Primer sequences of target genes.

Table S4. Summary of three-way ANOVA results on effects of nano-TiO_2_ and temperature.

Table S5. Summary of two-way ANOVA results.

Table S6. Summary of two-way ANOVA results.

Table S7. Summary of two-way ANOVA results.

Figure S1. The survival rate (A) and condition index (B) of *M. coruscus*.

Figure S2. The foot stained with hematoxylin and eosin (H&E).

Figure S3. The correlation analysis.

Figure S4. Pearson’s correlation coefficients.

Figure S5. Biplot from principal component analysis.

Reference

**Text S1. Methods for detection of titanium**

The surface morphology (size and shape) of nano-TiO₂ particles was characterized using scanning electron microscopy (SEM, KYKY-EM8000, China) and transmission electron microscopy (TEM, Hitachi HT-7700, Japan). Hydrodynamic diameter and zeta potential were determined at ambient sea surface temperature (22°C) and simulated marine heatwave condition (28°C) using a Malvern laser particle sizer (Mastersizer 2000, Malvern Instruments, UK), to assess nanoparticle dispersion stability (Hou et al., 2017; 2018).

Fresh mussel foot tissues (~200 mg per group on days 3, 10, and 13) were dried at 65°C for 24 h and subsequently digested in 5 mL of 65% nitric acid at 150°C. Complete digestion was achieved using a microwave digestion system (CEM, Matthews, NC, USA). Titanium concentrations in water samples and tissues were measured by inductively coupled plasma mass spectrometry (ICP-MS) and used as a proxy for TiO_2_ levels (Wei et al., 2025).

**Text S2. Determination of the adhesion of byssal threads**

All seawater in the tank was drained, and the mussel was positioned beneath the sensor assembly of the pressure gauge (Gu et al., 2023). After recording the number of byssal threads, a mussel was affixed to the sensor assembly of the pressure gauge via a traction rope. Subsequently, the mussel was gradually pulled at a constant speed of 10 mm/min (directly below the digital pressure gauge) until the byssal thread broke. The maximum force recorded on the dynamometer during this process was defined as adhesion strength of total byssal threads (Gu et al., 2023; Mackenzie et al., 2014). Then, we obtained the adhesion strength of single byssal threads by dividing the adhesion strength of total byssal threads by the number of byssal threads. All data collected during the measurement were automatically recorded and saved by the computer connected to the sensor.

**Text S3. Methods for histological observation of the mussel foot**

The foot samples of *M. coruscus* from each treatment group were fixed in Bouin’s Fixative (Guangzhou Lucheng Biological Technology Co., LTD, Guangzhou, China) for a 24-h period at 4°C. Then, the samples underwent a fixative replacement process involving 30%, 50%, and 70% ethanol baths for durations of 30, 20, and 20 min, respectively. Subsequently, the samples were subjected to dehydration through a graded series of ethanol concentrations (50%, 70%, 90%, and 100%), each for 45 min. After dehydration, the samples were sequentially exposed to a mixture of alcohol and xylene (1:1) for 40 min, two xylene baths for 15 min each, a mixture of xylene and paraffin wax (1:1) for 50 min at 60°C, and pure paraffin wax for 3 h at 60°C. Sections of the paraffin-embedded samples, with a thickness of 5 μm, were cut using a microtome (Leica, RM2245, Germany). For histological analysis, the sections were stained with Harris hematoxylin and eosin (BBI, Shanghai, China), covered with neutral balsam (Solarbio, Beijing, China) and photographed using the Ni-E microscope with a Nikon Ds-Ri2 camera (Nikon, Tokyo, Japan).

For histological analysis, the sections were stained with Harris hematoxylin and eosin (BBI, Shanghai, China), immersed for 5 min in 95% ethanol, twice for 5 min in 100% ethanol, and twice for 5 min in xylene. The sections were then covered with neutral balsam (Solarbio, Beijing, China) and photographed using the Ni-E microscope with a Nikon Ds-Ri2 camera (Nikon, Tokyo, Japan). The area of the foot glands was determined using FIJI software, and nine replicates were performed for each treatment group.

Grades 0 to 4 were assigned based on lesion extent as follows: Grade 0 indicated no abnormalities; Grade 1, lesions involving up to 25% of the foot; Grade 2, 25 < lesions ≤ 50%; Grade 3, 50 < lesions ≤ 75%; and Grade 4, more than 75% of the foot. The total histopathological score (HS) was calculated as the sum of the individual scores for each evaluated parameter, including the NBG and the LCE. The foot gland was with purplish red color and the muscles of foot were located at the periphery of the glands and were pink in color. The proportion of foot gland area versus foot was calculated.

**Text S4. Methods for the gene expression analysis**

The fresh foot was collected, washed with 1 × phosphate buffered saline (PBS) (pH = 7.4), and frozen at −80°C for further analysis. Total RNA was extracted with RNA Isolater Total RNA Extraction Reagent according to manufacturer’s instructions (Vazyme Biotech Co., Ltd.). After gel electrophoresis and OD260/280 assays (microspectrophotometer NanoDrop 2000, Thermo Scientific), total RNA of mussel foot was reverse transcribed to first strand cDNA with a HiScript III 1st Strand cDNA Synthesis Kit (+gDNA wiper) (Vazyme Biotech Co., Ltd).

Then, qRT-PCR was conducted with an ABI QuantStudio 6 Flex system (USA) using SYBR Green Master Mix (Vazyme Biotech Co., Ltd.). The following procedure was adopted for the amplification: 95°C predenaturation, 3 min; 95°C denaturation, 30 s; 56°C annealing, 30 s; 72°C extension, 1 min; 34 cycles; 72°C extension, 10 min. All primers were designed with Primer Premier 6.0 and synthesized by Sangon Biotech (Shanghai, China).

Quantitative real-time PCR was conducted on an ABI QuantStudio 6 Flex (USA) instrument using Taq Pro Universal SYBR qPCR Master Mix kit (Vazyme Biotech Co., Ltd). Following the procedure of Livak and Schmittgen (2001), the relative mRNA expression level of each target gene was calculated with the 2^−ΔΔCT^ method with *18S* rRNA as the internal reference.

**Text S5. Methods for the energy-related, oxidative and immune-related parameters in the foot**

Foot tissue (0.1g) were homogenized in 0.9 mL 1 × PBS (pH = 7.4, 4°C) at 4°C with Teflon Potter-Elvehjem homogenizers (CEBO-64, China), and centrifuged at 4°C and 8,000 x g for 10 min. The protein concentrations were measured by Coomassie Brilliant Blue (G-250) method according to Bradford (1976) using a commercial kit (A045-2, Nanjing Jiancheng Bioengineering Institute, China) with bovine serum albumin (BSA) as a standard.

Glucose content (A154-1) was measured using the glucose oxidase method: samples were mixed with a working solution containing glucose oxidase, peroxidase, and 4-aminoantipyrine, incubated at 28°C for 10 min, and absorbance was read at 505 nm. Triglycerides (TG; A110-1) were assessed by the Glycerol-3-Phosphate Oxidase – Phenol 4-Aminoantipyrine Peroxidase (GPO-PAP) method, where samples were incubated with a solution containing Tris-HCl, ATP, lipase, and glycerol kinase at 28°C for 10 min and measured at 510 nm. Albumin (ALB; A028-2) content was determined by its reaction with bromocresol green, producing a color change from yellow to green, with absorbance read at 630 nm after incubation at room temperature for 10 min.

Superoxide dismutase (SOD; A001-3) activity was measured according to the kit instructions. Catalase (CAT; A007-1-1) activity was determined by monitoring the initial rate of decrease in absorbance at 405 nm using the ammonium molybdate method. Glutathione peroxidase (GPX; A005-1) activity was assessed following the manufacturer's protocol. Lipid peroxidation (LPO; A106-1) levels were evaluated by measuring the absorbance of the chromophore formed with a chromogenic reagent at 586 nm, reflecting lipid peroxidation levels (Ohkawa et al., 1979).

Activities of acid phosphatase (ACP; A060-2) and alkaline phosphatase (AKP; A059-2) were measured using disodium phenyl phosphate as substrate, with absorbance read at 520 nm. All enzyme assays were conducted at 28°C. All assays were performed using a microplate reader (Flexstation®3, Molecular Devices, CA, USA) with kits supplied by Nanjing Jiancheng Bioengineering Institute, China.

**Text S6. Methods for of cell viability and reactive oxygen species assays**

Foot cells were isolated from mussels following established protocols with some modifications. Initially, approximately 500 mg of fresh foot tissues were finely cut using sterilized scissors in 1 mL of 1 × PBS (pH = 7.4, 4°C). After centrifugation at 1000 × g for 1 min, the supernatant was discarded, and 1 mL of calcium- and magnesium-free saline (CMFS: 20 mM HEPES, 500 mM NaCl, 12.5 mM KCl, and 5 mM EDTA, pH 7.3). The mixture was then incubated at 28°C for 10 min. Following the incubation period, the sample was centrifuged at 1000 × g for 1 min, and the supernatant was discarded. The cells were washed with 1 × PBS (pH = 7.4, 4°C) and the process was repeated. Finally, the isolated cells were filtered through 100 μm nets.

To assess cell viability, 20 μL of CCK-8 solution (CCK-8; Beyotime Biotechnology, China). was added to 200 μL of cells suspension in 96-well plates. The control group: 200 μL of resuspended cells was centrifuged at 1000 × g for 5 min at 4°C, collecting the supernatant, and adding 20 μL of CCK-8 solution. Then, both foot cells and cell-free media samples were incubated for 1 h at 28°C and absorbance was measured at 450 nm with an automatic microplate reader (Flexstation®3, Molecular Devices, CA, USA).

For assessment of the apoptosis rates, 500 μL of cells suspension was centrifuged at 1000 × g, 4°C for 5 min and the supernatant was discarded. The cells were then gently resuspended in 195 μL of Annexin V-FITC binding buffer. Next, 5 μL Annexin V-FITC and 10 μL propidium iodide (PI) were sequentially added to the cell suspension tube with gentle mixing after each addition. The samples were incubated for 20 min at room temperature in the dark, and then placed in in ice-water mixture. Meanwhile, unstained, PI-stained and Annexin V-FITC single-stained hemocytes were prepared as negative controls. Fluorescence was measured using a flow cytometer (BD Accuri® C6, USA) equipped with an air-cooled argon laser (excitation at 448 nm). The frequency distribution histogram of fluorescence intensity of each sample was obtained. The number of Annexin V-FITC- and PI-positive cells as a percentage of the total number of cells represented the proportion of apoptotic cells.

For the assessment of ROS levels (Tang et al., 2020; Wu et al., 2018), DCFH-DA was diluted 1:1000 with 1× PBS (pH 7.4) to a final concentration of 10 μmol/L. A total of 5 μL of the diluted DCFH-DA was added to 500 μL of the cell suspension and incubated at 28°C for 20 min, with gentle inversion every 3 min to ensure thorough mixing. Fluorescence intensity was then measured using an automatic microplate reader (Synergy™ Neo2 Multi-Mode Microplate Reader) at an excitation wavelength of 488 nm and an emission wavelength of 525 nm. ROS content was expressed as arbitrary fluorescence units (A.U.) per approximately 2.5 × 10^6^ cells.

**Table S1 Measured concentrations of nano-TiO_2_ in experimental exposures (ND indicates not detected, N = 3). All data are expressed as concentrations of TiO_2_, calculated based on the Ti contents detected by ICP-MS.**

| Treatments | Exposure concentration measured |
| --- | --- |
|  | (μg/L) |
| 22ºC (control) | ND |
| 22ºC * 25 μg/L | 22.55 ± 0.87 |
| 22ºC * 250 μg/L | 235.74 ± 1.58 |
| MHWs | ND |
| 28ºC * 25 μg/L | 23.12 ± 1.94 |
| 28ºC * 250 μg/L | 238.63 ± 5.59 |

**Table S2 Seawater parameters (mean ± SD) in experimental exposures.**

Dissolved oxygen (DO, mg/L) and salinity (practical salinity units) were measured daily, and pH and temperature were continuously monitored during the experimental exposure. N = 13 for DO and salinity and N = 39 for pH and temperature.

| Exposure time  (day) | Treatments | Temperature  (°C) | DO  (mg/L) | Salinity | pH |
| --- | --- | --- | --- | --- | --- |
| Day 0 | Normal temperature  MHWs | 22.2 ± 0.2  22.1 ± 0.1 | 5.85 ± 0.3  6.03 ± 0.5 | 25.1 ± 0.22  25.3 ± 0.13 | 8.10 ± 0.03  8.11 ± 0.03 |
| Day 1 | Normal temperature  MHWs | 22.1 ± 0.1  24.3 ± 0.4 | 5.91 ± 0.5  5.87 ± 0.7 | 25.1 ± 0.17  25.1 ± 0.19 | 8.12 ± 0.02  8.11 ± 0.05 |
| Day 2 | Normal temperature  MHWs | 22.3 ± 0.3  26.2 ± 0.7 | 5.98 ± 0.4  5.95 ± 0.5 | 25.1 ± 0.13  25.2 ± 0.09 | 8.10 ± 0.02  8.11 ± 0.04 |
| Day 3 | Normal temperature  MHWs | 22.1 ± 0.4  28.2 ± 0.4 | 5.99 ± 0.8  5.97 ± 0.2 | 25.1 ± 0.08  25.1 ± 0.19 | 8.10 ± 0.03  8.12 ± 0.01 |
| Day 4-Day10 | Normal temperature  MHWs | 22.1 ± 0.3  28.3 ± 0.5 | 6.04 ± 0.2  5.88 ± 0.6 | 25.3 ± 0.11  25.1 ± 0.06 | 8.11 ± 0.01  8.11 ± 0.03 |
| Day 11 | Normal temperature  MHWs | 22.3 ± 0.1  26.4 ± 0.1 | 5.97 ± 0.6  5.87 ± 0.4 | 25.2 ± 0.03  25.1 ± 0.18 | 8.11 ± 0.02  8.11 ± 0.01 |
| Day 12 | Normal temperature  MHWs | 22.2 ± 0.3  24.5 ± 0.1 | 6.01 ± 0.8  5.89 ± 0.5 | 25.1 ± 0.14  25.4 ± 0.22 | 8.12 ± 0.03  8.12 ± 0.02 |
| Day 13 | Normal temperature  MHWs | 22.1 ± 0.5  22.3 ± 0.5 | 5.94 ± 0.7  5.97 ± 0.3 | 24.7 ± 0.15  25.2 ± 0.17 | 8.10 ± 0.03  8.11 ± 0.01 |

**Table S3 Primers used for qRT-PCR (56°C annealing temperature).**

| Primer name | Primer sequences (5'-3') |
| --- | --- |
| Internal reference *18S* rRNA *18S* | F: GAACTACGACGGTATCTGAT |
|  | R: CCTTGGTGCTCTTGATTGA |
| Heat shock-related genes *HSP-70* | F: GAAATTGTCTTAGTAGGTGGATC |
|  | R: CAGGCTGGTTGTCGGAGTAAGT |
| *HSP-90* | F: GGAAATCCGAATCATACCAGAC |
|  | R: ACCTCTGCCGATGGACTCAC |
| Adhesive-related genes *Mfp-1* | F: TGGCTACAATTCAAGAACTG |
|  | R: AGAGAAGGATGAGAACGAA |
| *Mfp-2* | F: CGGTCACAGAAGCATCAT |
|  | R: CATCCTCATCGTCGTCATAT |
| *Mfp-3* | F: TTTGCTGGCTTTAGTCCTT |
|  | R: ACCGCTATTCCATCCCTTA |
| *Mfp-4* | F: ACATATTCACAGCCACCAA |
|  | R: TCACCGTATGATTCAAGACA |
| *Mfp-5* | F: TTGGTGCTCGTTCTTGTAA |
|  | R: TGCTACTGCCTCCATAATG |
| *Mfp-6* | F: CGGTGATTATGATTACAGAGG |
|  | R: GAAGACAGCATCCAGCAT |
| Pre-collagens proteins-related genes *PreCOL-D* | F: ACCAAGAGGAGATAGAGGAG |
|  | R: GGCTGTTCTGAGGTCTTC |
| *PreCOL-P* | F: GATCTTCACATGCATCAGC |
|  | R: CACTGCCACCTCCTAAAC |
| *PreCOL-NG* | F: ACAAGGACCACAAGGAGAA |
|  | R: ACACCACCAACACCAGTT |
| Inflammation-related genes |  |
| *Caspase-3* | F: CCTGATCGTCGCTTTGTTGA |
|  | R: CTCTGGACAGTTCTCTGCATTG |
| *TLR-4* | F: GCCTGCACCACAAAGATTTCATACC |
|  | R: TTCCATTTGGCACCATTCACTTTCG |
| *Myd88* | F: GGTCCATTTCTCTCCAGGCTTTACG |
|  | R: TCCCACCATTTCAGCTAGTCCTCTC |
| *TNF-α* | F: AACCAACCGGTGATTGTGGT |
|  | R: TGGGATCAAGCAGCAACCAA |

**Table S4 Summary of three-way ANOVA results on effects of temperature (T), nano-TiO_2_ (TiO_2_), time and their interactions on the byssus traits and transcript levels of heat shock proteins in the foot.**

Number (NBT) and length of the byssus threads, adhesion of all byssal threads (ATBT) and of a single thread (ASBT), as well as mRNA expression of *HSP-70* and *HSP-90* were analysed as dependent variables.

|  | df | NBT | | | Length | | | ATBT | | |
| --- | --- | --- | --- | --- | --- | --- | --- | --- | --- | --- |
|  |  | MS | F | P | MS | F | P | MS | F | P |
| T | 1 | 13863.21 | 142.805 | <0.01 | 61.504 | 0.173 | 0.679 | 439.569 | 663.222 | <0.01 |
| TiO_2_ | 2 | 553.911 | 5.706 | <0.01 | 641.819 | 1.808 | 0.171 | 230.120 | 347.206 | <0.01 |
| Time | 2 | 38731.07 | 398.970 | <0.01 | 1511.979 | 4.258 | 0.018 | 296.674 | 447.623 | <0.01 |
| T* TiO_2_ | 2 | 1668.311 | 17.185 | <0.01 | 104.069 | .293 | 0.747 | 2.413 | 3.641 | 0.031 |
| T* Time | 2 | 4439.211 | 45.728 | <0.01 | 84.693 | 0.239 | 0.788 | 15.076 | 22.747 | <0.01 |
| TiO_2_* Time | 4 | 397.028 | 4.090 | <0.01 | 180.149 | 0.507 | 0.730 | 20.729 | 31.275 | <0.01 |
| T* TiO_2_* Time | 4 | 857.361 | 8.832 | <0.01 | 23.864 | 0.067 | 0.992 | 1.733 | 2.615 | 0.042 |
|  | df | ASBT | | | *HSP-70* | | | *HSP-90* | | |
|  |  | MS | F | P | MS | F | P | MS | F | P |
| T | 1 | 0.019 | 219.778 | <0.01 | 0.056 | 5.128 | <0.01 | 1.197 | 158.125 | <0.01 |
| TiO_2_ | 2 | 0.014 | 168.491 | <0.01 | 0.153 | 13.908 | <0.01 | 0.517 | 68.330 | <0.01 |
| Time | 2 | 0.002 | 19.950 | <0.01 | 2.699 | 245.310 | <0.01 | 7.952 | 1050.76 | <0.01 |
| T* TiO_2_ | 2 | 0.001 | 6.391 | <0.01 | 0.532 | 48.318 | <0.01 | 0.000 | 0.035 | 0.966 |
| T* Time | 2 | 0.001 | 12.763 | <0.01 | 12.190 | 1107.724 | <0.01 | 11.101 | 1467.02 | <0.01 |
| TiO_2_* Time | 4 | 0.000 | 4.335 | <0.01 | 0.432 | 39.295 | <0.01 | 0.150 | 19.834 | <0.01 |
| T* TiO_2_* Time | 4 | 0.000 | 1.504 | 0.210 | 0.863 | 78.454 | <0.01 | 0.224 | 29.601 | <0.01 |

**Table S5 Summary of three-way ANOVA results on effects of temperature (T), nano-TiO_2_ (TiO_2_), time and their interactions on the mRNA expression of byssus-related genes in the foot of *M. coruscus*.**

|  | *df* | *Mfp-1* | | | *Mfp-2* | | | *Mfp-3* | | | *Mfp-4* | | |  | *Mfp-5* |  |
| --- | --- | --- | --- | --- | --- | --- | --- | --- | --- | --- | --- | --- | --- | --- | --- | --- |
|  |  | MS | F | *P* | MS | F | *P* | MS | F | *P* | MS | F | *P* | MS | F | *P* |
| T | 1 | 0.633 | 22.829 | <0.01 | 0.005 | 0.455 | 0.502 | 31.777 | 384.803 | <0.01 | 5.503 | 323.140 | <0.01 | 63.685 | 299.266 | <0.01 |
| TiO_2_ | 2 | 0.569 | 20.511 | <0.01 | 0.079 | 7.486 | <0.01 | 4.031 | 48.815 | <0.01 | 0.596 | 34.976 | <0.01 | 50.906 | 239.216 | <0.01 |
| Time | 2 | 15.684 | 565.753 | <0.01 | 9.344 | 884.522 | <0.01 | 33.641 | 407.372 | <0.01 | 4.919 | 288.842 | <0.01 | 109.947 | 516.657 | <0.01 |
| T* TiO_2_ | 2 | 0.487 | 17.564 | <0.01 | 0.550 | 52.105 | <0.01 | 0.337 | 4.078 | 0.021 | 1.360 | 79.875 | <0.01 | 83.377 | 391.800 | <0.01 |
| T* Time | 2 | 13.116 | 473.119 | <0.01 | 0.964 | 91.235 | <0.01 | 7.075 | 85.675 | <0.01 | 1.676 | 98.428 | <0.01 | 23.379 | 109.863 | <0.01 |
| TiO_2_* Time | 4 | 0.462 | 16.648 | <0.01 | 0.492 | 46.587 | <0.01 | 1.810 | 21.918 | <0.01 | 0.551 | 32.336 | <0.01 | 17.240 | 81.011 | <0.01 |
| T* TiO_2_* Time | 4 | 0.399 | 14.397 | <0.01 | 0.493 | 46.703 | <0.01 | 0.906 | 10.977 | <0.01 | 0.746 | 43.809 | <0.01 | 14.594 | 68.578 | <0.01 |
|  | *df* | *Mfp-6* | | | *preCOL-D* | | | *preCOL-P* | | | *preCOL-NG* | | |  |  |  |
|  |  | MS | F | *P* | MS | F | *P* | MS | F | *P* | MS | F | *P* |  |  |  |
| T | 1 | 6.061 | 283.004 | <0.01 | 0.865 | 66.692 | <0.01 | 0.950 | 31.120 | <0.01 | 2.925 | 344.970 | <0.01 |  |  |  |
| TiO_2_ | 2 | 0.780 | 36.439 | <0.01 | 0.339 | 26.142 | <0.01 | 8.010 | 262.456 | <0.01 | 0.307 | 36.155 | <0.01 |  |  |  |
| Time | 2 | 9.667 | 451.384 | <0.01 | 21.643 | 1669.570 | <0.01 | 23.984 | 785.865 | <0.01 | 0.970 | 114.386 | <0.01 |  |  |  |
| T* TiO_2_ | 2 | 6.269 | 292.708 | <0.01 | 0.130 | 9.992 | <0.01 | 0.645 | 21.136 | <0.01 | 0.558 | 65.855 | <0.01 |  |  |  |
| T* Time | 2 | 0.592 | 27.623 | <0.01 | 4.991 | 385.011 | <0.01 | 1.800 | 58.983 | <0.01 | 0.127 | 14.958 | <0.01 |  |  |  |
| TiO_2_* Time | 4 | 0.217 | 10.140 | <0.01 | 2.145 | 165.454 | <0.01 | 3.104 | 101.707 | <0.01 | 0.119 | 14.050 | <0.01 |  |  |  |
| T* TiO_2_* Time | 4 | 0.743 | 34.696 | <0.01 | 0.334 | 25.793 | <0.01 | 1.468 | 48.091 | <0.01 | 0.209 | 24.605 | <0.01 |  |  |  |

**Table S6 Summary of three-way ANOVA results on effects of temperature (T), nano-TiO_2_ (TiO_2_), time and their interactions on the parameters related to biogenetics, redox and immune status, and cell viability in the foot of *M. coruscus*.**

|  | *df* | CV | | | CA | | | SOD | | | CAT | | | GPX | | | LPO | | |
| --- | --- | --- | --- | --- | --- | --- | --- | --- | --- | --- | --- | --- | --- | --- | --- | --- | --- | --- | --- |
|  |  | MS | F | *P* | MS | F | *P* | MS | F | *P* | MS | F | *P* | MS | F | *P* | MS | F | *P* |
| T | 1 | 168957.590 | 668.971 | <0.01 | 811.200 | 1395.007 | <0.01 | 755.999 | 85.305 | <0.01 | 2.235 | 11.771 | <0.01 | 807.393 | 44.072 | <0.01 | 11.384 | 1636.303 | <0.01 |
| TiO_2_ | 2 | 139262.995 | 551.398 | <0.01 | 146.120 | 251.279 | <0.01 | 1863.145 | 210.232 | <0.01 | 9.270 | 48.826 | <0.01 | 1477.476 | 80.648 | <0.01 | 1.908 | 274.211 | <0.01 |
| Time | 2 | 8311.257 | 32.908 | <0.01 | 19.729 | 33.927 | <0.01 | 1309.326 | 147.741 | <0.01 | 120.524 | 634.810 | <0.01 | 38819.744 | 2118.978 | <0.01 | 0.630 | 90.574 | <0.01 |
| T* TiO_2_ | 2 | 3007.701 | 11.909 | <0.01 | 34.092 | 58.628 | <0.01 | 377.435 | 42.589 | <0.01 | 52.541 | 276.740 | <0.01 | 799.501 | 43.641 | <0.01 | 0.020 | 2.938 | 0.059 |
| T* Time | 2 | 21653.189 | 85.734 | <0.01 | 33.510 | 57.627 | <0.01 | 1294.241 | 146.039 | <0.01 | 69.600 | 366.586 | <0.01 | 2597.721 | 141.797 | <0.01 | 0.015 | 2.162 | <0.01 |
| TiO_2_* Time | 4 | 1476.767 | 5.847 | <0.01 | 4.557 | 7.837 | <0.01 | 473.169 | 53.391 | <0.01 | 7.558 | 39.809 | <0.01 | 2000.081 | 109.175 | <0.01 | 0.045 | 6.533 | <0.01 |
| T* TiO_2_* Time | 4 | 1185.505 | 4.694 | <0.01 | 3.956 | 6.803 | <0.01 | 340.990 | 38.476 | <0.01 | 4.647 | 24.476 | <0.01 | 2526.041 | 137.884 | <0.01 | 0.112 | 16.048 | <0.01 |
|  | *df* | ROS | | | ACP | | | AKP | | | Glu | | | TG | | | ALB | | |
|  |  | MS | F | *P* | MS | F | *P* | MS | F | *P* | MS | F | *P* | MS | F | *P* | MS | F | *P* |
| T | 1 | 775808.178 | 2069.95 | <0.01 | 0.808 | 493.621 | <0.01 | 0.868 | 291.749 | <0.01 | 0.173 | 136.485 | <0.01 | 1.630 | 1856.287 | <0.01 | 16.760 | 440.813 | <0.01 |
| TiO_2_ | 2 | 220740.433 | 588.964 | <0.01 | 0.124 | 75.516 | <0.01 | 0.358 | 120.362 | <0.01 | 0.012 | 9.458 | <0.01 | 0.920 | 1047.738 | <0.01 | 4.785 | 125.863 | <0.01 |
| Time | 2 | 3917.433 | 10.452 | <0.01 | 0.159 | 96.987 | <0.01 | 1.801 | 605.108 | <0.01 | 0.439 | 346.935 | <0.01 | 0.102 | 116.096 | <0.01 | 12.877 | 338.696 | <0.01 |
| T* TiO_2_ | 2 | 16820.344 | 44.879 | <0.01 | 0.083 | 50.789 | <0.01 | 1.069 | 359.267 | <0.01 | 0.352 | 277.753 | <0.01 | 0.041 | 46.309 | <0.01 | 0.487 | 12.810 | <0.01 |
| T* Time | 2 | 46725.211 | 124.669 | <0.01 | 0.092 | 56.195 | <0.01 | 0.027 | 9.170 | <0.01 | 0.425 | 335.664 | <0.01 | 0.017 | 19.427 | <0.01 | 0.318 | 8.369 | <0.01 |
| TiO_2_* Time | 4 | 2261.417 | 6.034 | <0.01 | 0.041 | 25.339 | <0.01 | 0.157 | 52.637 | <0.01 | 0.008 | 6.310 | <0.01 | 0.054 | 61.767 | <0.01 | 0.065 | 1.716 | 0.156 |
| T* TiO_2_* Time | 4 | 2403.628 | 6.413 | <0.01 | 0.029 | 17.613 | <0.01 | 0.187 | 62.901 | <0.01 | 0.049 | 38.961 | <0.01 | 0.034 | 38.790 | <0.01 | 2.389 | 62.824 | <0.01 |

**Table S7 Summary of three-way ANOVA results on effects of temperature (T), nano-TiO_2_ (TiO_2_), time and their interactions on the mRNA expression of apoptosis, inflammation and immune-related genes in the foot of *M. coruscus*.**

|  | *df* | *Caspase-3* | | | *TLR-4* | | | *Myd88* | | | *TNF-α* | | |
| --- | --- | --- | --- | --- | --- | --- | --- | --- | --- | --- | --- | --- | --- |
|  |  | MS | F | *P* | MS | F | *P* | MS | F | *P* | MS | F | *P* |
| T | 1 | 16.743 | 2142.952 | <0.01 | 8.151 | 641.237 | <0.01 | 16.150 | 3974.588 | <0.01 | 22.893 | 1723.224 | <0.01 |
| TiO_2_ | 2 | 3.225 | 412.796 | <0.01 | 0.939 | 73.873 | <0.01 | 2.511 | 618.023 | <0.01 | 5.456 | 410.649 | <0.01 |
| Time | 2 | 0.487 | 62.278 | <0.01 | 0.307 | 24.162 | <0.01 | 0.267 | 65.677 | <0.01 | 0.272 | 20.462 | <0.01 |
| T* TiO_2_ | 2 | 0.133 | 17.010 | <0.01 | 0.368 | 28.939 | <0.01 | 0.001 | 0.235 | 0.792 | 1.075 | 80.886 | <0.01 |
| T* Time | 2 | 0.602 | 77.065 | <0.01 | 0.123 | 9.674 | <0.01 | 0.069 | 17.042 | <0.01 | 0.182 | 13.679 | <0.01 |
| TiO_2_* Time | 4 | 0.077 | 9.916 | <0.01 | 0.056 | 4.404 | <0.01 | 0.050 | 12.387 | <0.01 | 0.073 | 5.500 | <0.01 |
| T* TiO_2_* Time | 4 | 0.076 | 9.669 | <0.01 | 0.108 | 8.488 | <0.01 | 0.052 | 12.834 | <0.01 | 0.073 | 5.508 | <0.01 |

**Figure S1** The survival rate (A) and condition index (B) of *M. coruscus* under nano-TiO_2_ (0, 25, and 250 μg/L) and temperature (22℃ and MHWs) conditions after 13-day exposure. Different small letters indicate significant difference between 0, 25, and 250 μg/L nano-TiO_2_ treatments (*P* < 0.05). Asterisk (*) indicates a significant difference between two temperature regimes (**P* < 0.05, ***P* < 0.01).

**Figure S2** Foot tissue of *M. coruscus* stained with hematoxylin and eosin (H&E) after 13-day exposure: control (A), 25 μg/L nano-TiO₂ at 22°C (B), 250 μg/L nano-TiO₂ at 22°C (C), MHWs (D), 25 μg/L nano-TiO₂ under MHWs (E), and 250 μg/L nano-TiO₂ under MHWs (F). (G) Percentage of foot gland area under different nano-TiO₂ (0, 25, and 250 μg/L) and temperature conditions (22°C and MHWs). Different lowercase letters indicate significant differences among nano-TiO₂ treatments (*P* < 0.05). Asterisks (*) denote significant differences between temperature regimes (**P* < 0.05, ***P* < 0.01).

**Figure S3** The correlation analysis between adhesion of the total byssal threads and number of byssal thread (A), *Mfp-3* (B), *Mfp-5* (C), and *Mfp-6* (D) of *M. coruscus* under nano-TiO_2_ (0, 25, and 250 μg/L) and temperature (22℃ and MHWs) conditions after 13-day exposure.

**Figure S4** Pearson’s correlation coefficients of measured variables at day 3, day 10, and day 13.

**Figure S5** Principal component analysis (PCA) biplots of measured variables at days 3, 10, and 13. (A) PCA based on gene expression levels and byssal thread characteristics.(B) PCA based on physiological and biochemical indices..

**References**

Bradford, M.M., 1976. A rapid and sensitive method for the quantitation of microgram quantities of protein utilizing the principle of protein-dye binding. Analytical Biochemistry, 72, 248–254.

Gu, H.X., Wei, S.S., Tu, Z.H., Hu, M.H., Guo, B.Y., Wang, Y.J., 2023. Polybrominated diphenyl ether-47 and food shortage impair the byssal attachment and health of marine mussels. Science of the Total Environment, 891, 164415.

Hou, J., Liu, H., Wang, L., Duan, L., Li, S., Wang, X., 2018. Molecular toxicity of metal oxide nanoparticles in *Danio rerio*. Environmental Science & Technology, 52, 7996−8004.

Hou, J., Zhou, Y., Wang, C., Li, S., Wang, X., 2017. Toxic effects and molecular mechanism of different types of silver nanoparticles to the aquatic crustacean *Daphnia magna*. Environmental Science & Technology, 51, 12868−12878.

Livak, K.J., Schmittgen, T.D., 2001. Analysis of relative gene expression data using real-time quantitative PCR and the 2^-ΔΔCT^ Method. Methods, 25, 402–408.

Mackenzie, C.L., Lynch, S.A., Culloty, S.C., Malham, S.K., 2014. Future oceanic warming and acidification alter immune response and disease status in a commercial shellfish species, *Mytilus edulis* L. PLoS One, 9, 99712.

Ohkawa, H., Ohishi, N., Yagi, K., 1979. Assay for lipid peroxides in animal tissues bythiobarbituric acid reaction. Analytical Biochemistry, 95, 351–358.

Tang, Y., Rong, J.H, Guan, X.F, Zha, S.J, Shi, W., Han, Y., Du, X.Y., Wu, F.Z., Huang, W., Liu, G.X., 2020. Immunotoxicity of microplastics and two persistent organic pollutants alone or in combination to a bivalve species. Environmental Pollution, 258, 113845.

Wei, S.S., Hu, M.H., Sokolova, I., Tu, Z.H., Chen, L.M., Xu, P., Mao, Y.R., Wang, S.X., Wang, Y.J., 2025. Laboratory-simulated marine heatwave enhances physiological damage to mussels exposed to titanium dioxide nanoparticles by disrupting the gut-hepatopancreas axis. Journal of Hazardous Materials, 486, 137006.

Wu, H.F., Zhong, M.Y., Lu, Z., Shan, X.J., Li, F., Ji, C.L., Cong, M., 2018. Biological effects of tris (1-chloro-2-propyl) phosphate (TCPP) on immunity in mussel *Mytilus galloprovincialis*. Environmental Toxicology and Pharmacology, 61, 102–106.
